# Biomaterial-Based Nucleic Acid Delivery Systems for In Situ Tissue Engineering and Regenerative Medicine

**DOI:** 10.3390/ijms26157384

**Published:** 2025-07-30

**Authors:** Qi-Xiang Wu, Natalia De Isla, Lei Zhang

**Affiliations:** 1 Medical School, Kunming University of Science and Technology, Kunming 650032, China; kgyxywqx@163.com; 2 Biopôle of Medical School, University of Lorraine, 54000 Vandoeuvre-Lès-Nancy, France; natalia.de-isla@univ-lorraine.fr; 3 College of Medicine, Southwest Jiaotong University, Chengdu 610032, China; 4 Institute of Biomedical Engineering, Southwest Jiaotong University, Chengdu 610032, China

**Keywords:** nucleic acid delivery system, gene therapy, vector, nanoparticle, scaffold

## Abstract

Gene therapy is a groundbreaking strategy in regenerative medicine, enabling precise cellular behavior modulation for tissue repair. In situ nucleic acid delivery systems aim to directly deliver nucleic acids to target cells or tissues to realize localized genetic reprogramming and avoid issues like donor cell dependency and immune rejection. The key to success relies on biomaterial-engineered delivery platforms that ensure tissue-specific targeting and efficient intracellular transport. Viral vectors and non-viral carriers are strategically modified to enhance nucleic acid stability and cellular uptake, and integrate them into injectable or 3D-printed scaffolds. These scaffolds not only control nucleic acid release but also mimic native extracellular microenvironments to support stem cell recruitment and tissue regeneration. This review explores three key aspects: the mechanisms of gene editing in tissue repair; advancements in viral and non-viral vector engineering; and innovations in biomaterial scaffolds, including stimuli-responsive hydrogels and 3D-printed matrices. We evaluate scaffold fabrication methodologies, nucleic acid loading–release kinetics, and their biological impacts. Despite progress in spatiotemporal gene delivery control, challenges remain in balancing vector biocompatibility, manufacturing scalability, and long-term safety. Future research should focus on multifunctional “smart” scaffolds with CRISPR-based editing tools, multi-stimuli responsiveness, and patient-specific designs. This work systematically integrates the latest methodological advances, outlines actionable strategies for future investigations and advances clinical translation perspectives beyond the existing literature.

## 1. Introduction

Regenerative medicine focuses on efficient therapies to repair damaged or aging tissues and organs in the human body to restore their functions [1]. Current regenerative medicine therapies primarily employ stem cell-based approaches and scaffold-driven strategies. Stem cell transplantation has advanced significantly, with mesenchymal stem cells (MSCs) and stem cell sheets leveraged for their paracrine effects and regenerative potential [2]. Despite their therapeutic promise, clinical translation faces challenges including suboptimal harvesting protocols, stringent quality control requirements, low engraftment rates (<20%), rapid clearance, and inefficient delivery systems [2,3]. Scaffold-based approaches utilize biomaterials to provide structural and biochemical cues for repair. However, conventional scaffolds remain limited by poor drug release kinetics and inability to overcome biological barriers like endosomal escape for nucleic acids [4]. To address these limitations, researchers focus on developing cell-free approaches, with nucleic acid therapies gaining prominence as a versatile alternative.

The global novel COVID-19 pandemic has greatly accelerated the development of mRNA technology [5]. The development of various nucleic acid drugs has become a research hotspot, involving vaccines, tumor immunotherapy, cell reprogramming and other fields [6,7,8]. DNA-based nucleic acid therapy in regenerative medicine mainly includes the delivery of gene fragments and in vivo gene editing technology. In RNA-based nucleic acid therapy, small nucleic acids are the most commonly used, such as small interfering RNA (siRNA), microRNA (miRNA), and short hairpin RNA (shRNA). However, DNA and RNA therapy are impeded because of limitations such as enzymatic susceptibility, cellular barriers, passive accumulation in off-target organs, and high clearance [9]. Effective delivery systems must therefore protect nucleic acids from biological barriers while ensuring precise spatiotemporal release. Biomaterial-based nucleic acid delivery holds transformative potential for regenerative medicine. By integrating nucleic acids with precisely engineered biodegradable nanoparticles, stimuli-responsive scaffolds, and 3D-printed matrices, this strategy offers a promising solution to overcoming current barriers in spatiotemporal control, transfection efficiency, and safety—ultimately translating genetic instructions into programmable tissue regeneration. Current strategies—such as exosome encapsulation, nanoparticle formulations, and chemical or ligand modifications—show promise but require further optimization to balance efficiency with biocompatibility.

Biomaterial-based scaffolds represent a transformative approach to nucleic acid delivery, merging programmable gene therapies with bioinspired material design. These customizable platforms—deployable as 3D matrices or injectable formulations tailored to anatomical sites—address core limitations of standalone therapies through engineered control over degradation kinetics, nucleic acid dosing, and target gene selection. Critically, their clinical translation hinges on resolving long-term biomaterial safety profile [4].

Despite growing interest, a systematic analysis of scaffold design principles for nucleic acid delivery in tissue regeneration remains underexplored. This review bridges this gap by comprehensively evaluating cell-free nucleic acid delivery systems—from in vitro preparation to in vivo therapeutic applications—while highlighting key challenges and future directions in translational regenerative medicine.

## 2. Nucleic Acid Modification in Regenerative Medicine

**Figure 1:**
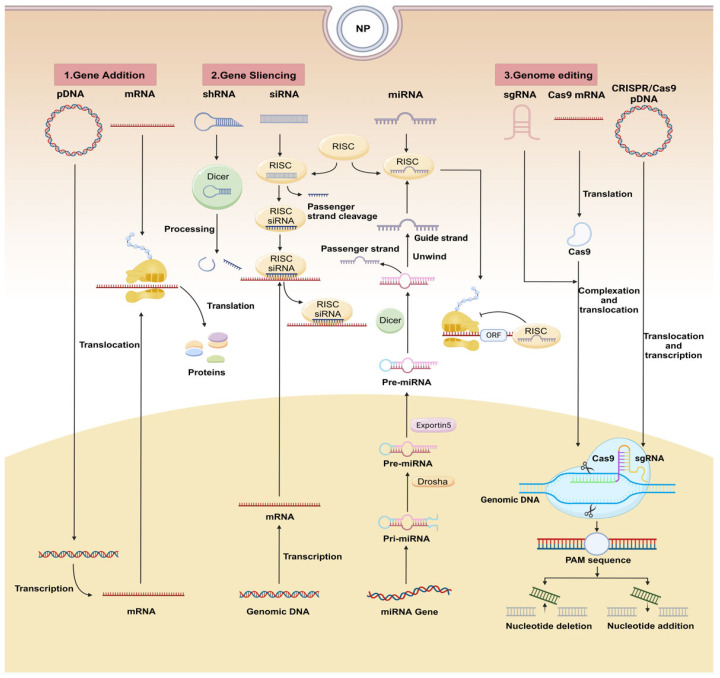
Three mechanisms of gene modification: gene addition, silencing, and CRISPR/Cas9-based genome editing (Created with Cnsknowall.com).

Nucleic acid-based therapeutics can precisely modulate stem cell functions by specifically targeting DNA or RNA molecules, thus promoting tissue regeneration. The mechanisms of commonly used nucleic acids can be categorized into three types: (1) gene addition, (2) gene silencing, and (3) genome editing. The specific mechanism is shown in Figure 1.

### 2.1. Gene Addition

The strategy of gene addition involves two primary approaches: plasmid DNA (pDNA) and mRNA. pDNA is a small, double-stranded circular DNA molecule, that can be transfected into the host cells via viral vectors or non-viral vectors. Once in cells, it can be integrated into the host genome or maintained as an extrachromosomal element, respectively [10,11]. mRNA, on the other hand, is synthesized through in vitro transcription. Unlike pDNA, exogenous mRNAs directly participate in gene expression without integrating into the host cell genome, thereby avoiding permanent genetic alterations [12]. In practical applications, selecting appropriate genes and optimizing delivery methods tailored to the target tissue and cell types are critical for achieving high-level expression of specific proteins.

### 2.2. Gene Silencing

Gene silencing strategies primarily operate through RNA interfering (RNAi), including mechanisms mediated by miRNAs (microRNA), siRNAs or shRNAs (small hairpin RNA).

Mature miRNA combined with RNA-induced silencing complex (RISC) and mainly binds to the 3′ untranslated region (3′UTR) of the targeted transcripts, enabling RNAi at the post-transcriptional level [13]. miRNAs have been shown to regulate important cellular processes such as migration, proliferation, and differentiation, especially in stem/progenitor cells [14,15,16]. Their expression exhibits cell- and tissue-type specificity. For example, miRNA-449c-3p is specifically expressed in spleen tissue, while miRNA-449a is enriched in lung, kidney and brain [17]. Notably, a single type of miRNA may regulate hundreds of gene targets, and an individual gene may be regulated by multiple miRNAs, resulting in cumulative and diverse biological effects [18]. Therefore, it is important to select appropriate miRNAs for therapeutic application.

By contrast, siRNAs specifically bind to target mRNAs via complementary base pairing and guide RISC (RNA-induced silencing complex) to cleave the target mRNA and suppress gene expression [19]. siRNA can modulate the expression of activators and inhibitors within specific pathways simultaneously, which makes them powerful tools for eliminating factors that inhibiting regeneration [20]. Due to their character of easy synthesis and chemical modification, siRNAs hold significant therapeutic potential.

Sharing functional similarities with siRNAs, shRNAs feature a hairpin structure. These molecules are cleaved by Dicer in the cytoplasm and converted into siRNA to execute gene silencing function [21].

### 2.3. Genome Editing with CRISPR/Cas9

The CRISPR/Cas9 system enables precise genome editing by forming a ribonucleoprotein complex composed of the Cas9 enzyme and a single-guide RNA (sgRNA). This complex creates specific double-stranded breaks in DNA, which are subsequently repaired by endogenous cellular repair mechanisms to achieve targeted genetic modifications. There are three forms of delivery vectors employed by the CRISPR/Cas9 system. The first is pDNA which encodes both Cas9 and sgRNA. The second is Cas9 mRNA and sgRNA, which are translated into functional Cas9 proteins in the cytoplasm. The third is direct delivery of RNPs (ribonucleoprotein complexes), which bound Cas9 protein to sgRNA. Among the three mechanisms, RNPs offer the quickest pathway. Beyond generating targeted gene edits, CRISPR/Cas9 can also activate or inhibit specific genes. In regenerative medicine, this technology serves two important roles. On the one hand, it can correct regeneration-related genetic defects or activate pre-programmed pathways to directly guide stem cell reprogramming. On the other, it can rejuvenate aged or differentiated stem cells by restoring their “stemness” and regenerative capacity [22]. This addresses the natural decline in stem cell potency that occurs as cells differentiate into somatic lineages, losing their ability to self-renew and repair tissues.

Above all, each of these strategies holds distinct therapeutic potential. While the CRISPR/Cas9-based genome editing system currently has limited applications in in situ tissue engineering, its precision and versatility position it as a highly promising tool for future advancements. Apart from these approaches, lncRNAs (long non-coding RNAs) also exhibit significant promise due to unique regulatory mechanisms, although their therapeutic applications remain unexplored and need further investigation [23,24].

## 3. Nucleic Acid Drug Delivery in Multi-Scale Tissue Engineering and Regenerative Medicine

**Figure 2:**
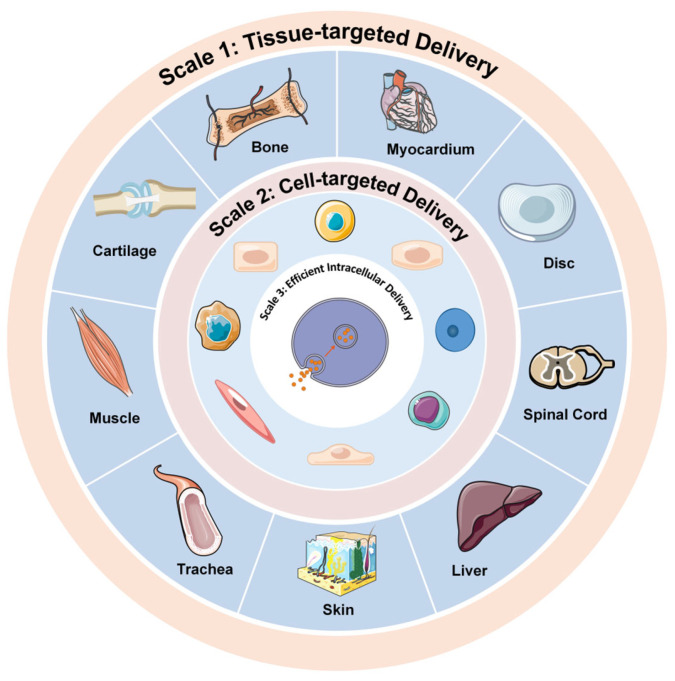
Three key scales of nucleic acid therapeutics for in situ tissue regeneration: tissue targeting, cellular specificity, and intracellular transport.

Despite their diverse mechanisms of action, nucleic acids share a common initial challenge, requiring the sequential achievement of three critical delivery steps: tissue-specific targeting, cellular recognition, and efficient intracellular transport (Figure 2).

### 3.1. Tissue-Targeted Delivery

Tissue-targeted delivery offers several therapeutic advantages by addressing site-specific challenges. In the knee joint, dynamic biomechanical loading during physical activity accelerates drug clearance and creates a heterogeneous distribution. Synovial fluid further reduces local drug concentrations through mechanical washout. To mitigate these limitations, bio-adhesive elastomers like hyaluronic acid-based hydrogels enable shear-resistant encapsulation of nucleic acid therapeutics, sustaining localized release while withstanding joint movement stresses [25]. Conversely, off-target tissue distribution of nucleic acids may not only decrease therapeutic efficacy by insufficient drug accumulation at the target site, but also risks systemic toxicity due to non-specific drug accumulation in non-target tissues.

Natural hydrogels, such as collagen or chitosan, possess excellent biocompatibility and closely resemble the extracellular matrix. However, this type of hydrogel is limited in mechanical strength. In contrast, synthetic polymer hydrogels provide highly tunable mechanical properties, but the inherent bio-inertness necessitates the incorporation of bioactive molecules to facilitate effective cell interaction. Hybrid hydrogels, such as chitosan/poly(vinyl alcohol) can combine the above advantages, such as controllable degradation kinetics, enhanced mechanical strength, and inherent antibacterial activity, albeit with potentially more complex fabrication processes [26].

### 3.2. Cell-Targeted Delivery

Following tissue-specific delivery, cellular targeting presents the next critical challenge for intracellular delivery. Precise cellular recognition is essential to maximize therapeutic outcomes. Current approaches utilize vectors functionalized with antibodies, peptides, or other ligands that bind target cell receptors, enabling receptor-mediated endocytosis [27,28]. Although effective in immune cell activation and oncology [28], these strategies remain underexplored in regenerative medicine. This gap underscores the need for tailored ligand–receptor systems targeting regenerative cell types (e.g., stem cells, fibroblasts). Recent advances in receptor-mediated delivery, exemplified by DUPA-conjugated miR-34a (2-[3-(1,3-dicarboxypropyl)ureido] pentanedioic acid), demonstrate how ligand modification enhances miRNA stability and cellular specificity [29]. Significantly, Purdue Institute researchers observed that folate-conjugated miR-34a failed in prostate cancer due to insufficient receptor expression, which highlight the critical importance of matching ligands to target cell biomarkers [30]. These insights provide strategic principles for developing precision mRNA therapies beyond oncology, particularly for regenerative applications requiring cell-type-specific delivery mediated by ligand–receptor systems.

### 3.3. Efficient Intracellular Delivery

Intracellular nucleic acid delivery faces three primary biological barriers. First, nuclease degradation necessitates encapsulation in protective nanocarriers (e.g., lipid nanoparticles) to shield against enzymatic cleavage [31]. Second, cell membrane impermeability arises from nucleic acids’ large molecular size and negative charge cell [32], hindering passive diffusion across anionic membranes. Cationic delivery systems (e.g., polymer–nucleic acid complexes) neutralize surface charge via electrostatic interactions, promoting cellular uptake [33]. Third, endosomal entrapment risks degradation in acidic lysosomes [34,35]. Escape strategies disrupt endosomal membranes through mechanisms like the “proton sponge effect,” where cationic polymers (e.g., polyethyleneimine, PEI) buffer endosomal pH, inducing osmotic swelling and membrane rupture [36].

Overcoming these barriers requires multifaceted approaches: optimizing nanocarrier biocompatibility and targeting specificity by mimicking tissue-specific mechanochemical properties; integrating stimuli-responsive systems (pH/enzyme/light-activated); and advancing interdisciplinary collaborations across materials science, molecular biology, and clinical medicine. These innovations will enable spatiotemporally controlled delivery with minimal off-target effects.

## 4. Vectors for Nucleic Acids

To address the above challenges and realize efficient intracellular delivery, researchers have developed viral vectors and non-viral vectors. Viral vectors exhibit high transfection efficiency, but have limitations, such as poor replication efficiency, limited gene-loading capacity, and the risk of triggering immune responses. In contrast, non-viral vectors, like nanocarriers, offer numerous attractive properties, such as low immunogenicity, robust loading capacity, structural stability, and ease of modification [37]. These properties make them a promising alternative for targeted delivery applications.

### 4.1. Overview of Vector Design

**Figure 3:**
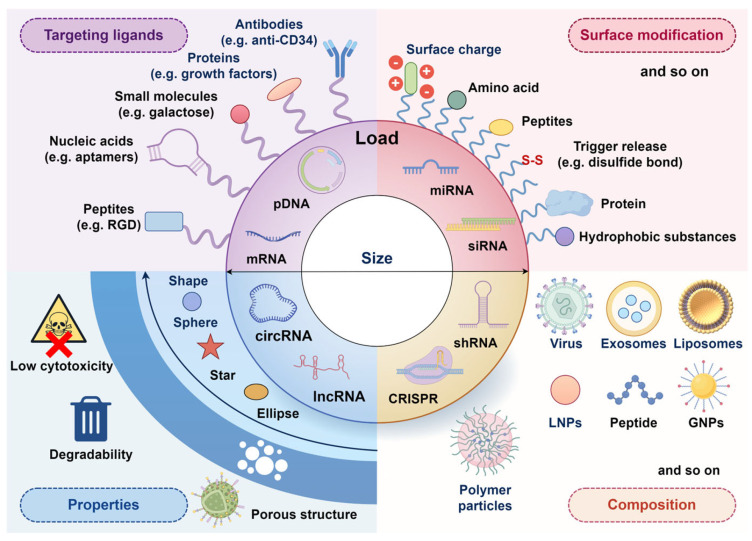
Schematics of targeting ligands, surface modification, physical properties and materials for nucleic acid delivery vectors. Created with Figdraw (https://www.figdraw.com).

Efficient intracellular delivery of nucleic acids relies on the tailored design of vectors (Figure 3). Nano-vectors can be of any shape and sometimes have a porous structure and are engineered with low cytotoxicity and degradable properties. Surface modifications of the carriers further enhance their functionality, improving nucleic acids, release mechanisms and cellular uptake. For instance, cationic or amino group-modified vectors can effectively penetrate the negatively charged cell membrane, while hydrophobic modifications facilitate traversal of the lipid bilayer.

Adenovirus vectors normally enter cells through coxsackie adenovirus receptors (CARs), which are inconsistently expressed in quiescent or proliferating cells, which results in adenovirus limited targeting specificity. To address this, Williams et al. have modified the adenoviral knob protein with basic fibroblast growth factor (FGF2), guiding adenovirus vectors to enter cells via fibroblast growth factor receptors (FGFRs) instead of CARs. FGFRs are expressed during normal and pathological tissue repair, but absent in quiescent cells. Subsequent studies combined this approach with the collagen matrix to deliver an adenoviral vector encoding platelet-derived growth factor-B, which has promoted wound healing, reduced toxicity, improved specificity and increased therapeutic potency [38]. Stimuli-responsive nanocarriers enable intelligent nucleic acid release in response to the tissue microenvironment. For example, miRNA conjugated to DSPE-PEG(1,2-distearoyl-sn-glycero-3-phosphoethanolamine-N-[methoxy(polyethylene glycol)]) via a disulfide bond, forming DSPE-PEG-miRNA. This linkage allows miRNA to be released intracellularly through disulfide bond cleavage triggered by glutathione (GSH). Additionally, surface modification with TAT (transactivator of transcription) peptide, a cell-penetrating peptide, enhances nanoparticle uptake in cardiovascular cells [39]. Furthermore, nanoparticle delivery capability can be improved by modification with targeting moieties, such as antibodies, nucleic acid aptamers, proteins, peptides, and some small molecules. For instance, mesoporous silica nanoparticles (MSN) coated with PEI and a layer of pentapeptide will specifically recognize and target delivery miR-146a-5p to endothelial cells [40]. Although stimuli-responsive and targeted delivery show great promise, they are still at a developmental bottleneck. Despite the promise of stimuli-responsive and targeted systems, challenges such as scalability, biocompatibility, and precision in controlled release remain significant developmental bottlenecks.

### 4.2. Advantages and Limitations of Natural Vectors

#### 4.2.1. Viral Vectors in Regenerative Medicine

Viral vectors such as adenovirus (AV), adeno-associated virus (AAV) and lentivirus (LV) are widely employed in regenerative medicine to deliver therapeutic genes that promote cell regeneration, tissue repair and functional recovery.

- Adenovirus

AVs have advantages of high efficiency, low pathogenicity and high viral titers. And they do not integrate into the host cell genome in vivo, making them suitable for transient gene expression. These vectors efficiently package DNA fragments into infectious viral particles, which mediate DNA delivery and expression in cells. While adenoviruses are widely used in gene therapy, safety concerns, particularly dose-dependent toxicity and immune response, require careful evaluation before clinical practice.

- Adeno-associated virus

Adeno-associated virus exhibits lower immunogenicity compared to adenovirus, especially recombinant AAV (rAAV). By removing the Rep and Cap genes and retaining the inverted terminal repeats of the virus, rAAV vectors safely package and carry foreign genes [41]. The genome of rAAV is naturally in the form of single-stranded DNA, which is converted to a double-stranded form by host cell DNA polymerase and subsequently transferred to the nucleus [42]. In the nucleus, rAAV genome entries targeted cells without integration into the host’s genome and it keeps the form of free, thereby minimizing the risk of insertional mutagenesis [43,44]. Also, AAV has a variety of serotypes, each of which has diverse tissue tropisms, providing a range of options and allowing tissue-specific targeting. For example, AAV6 is tropic to nerve cells, while AAV9 is tropic to cardiomyocytes [45,46]. AAV’s ability to cross the blood–brain barrier and transduce to skeletal muscle cells and cardiomyocytes with higher efficiency makes it a promising candidate for gene therapy in regenerative medicine [47].

- Lentivirus

Lentiviral vectors are gene transfer tools, derived from modified human immunodeficiency virus (HIV) or other lentiviral viruses. They are produced and assembled in packaging cells and released from cell membranes by budding. Once they enter the host cell, the LV RNA would be transcribed into DNA, which integrate transgenes into the host genome via reverse transcriptase and integrase enzymes. Lentiviral vectors can effectively infect both dividing and non-dividing cells, such as neurons and muscle cells. Through genetic engineering, LVs can target-specific cell subpopulations, such as astrocytes, to address localized cellular dysfunction without affecting other cells [48]. Also, the capacity of LV to accommodate relatively large transgenes is better than AAVs. Although the safety of lentiviral vectors has gradually improved, the immunogenicity and unpredictable integration sites remain challenges requiring further optimization.

#### 4.2.2. Non-Viral Vectors

Non-viral vectors, like exosome, lipid-based systems, peptide-based gene delivery systems, polysaccharide derivatives, and nucleic acid-based nanomaterials, have become a research hotspot due to their low immunogenicity and engineerable design. However, they commonly face challenges such as low transfection efficiency or inadequate stability in vivo, necessitating surface modifications (e.g., PEGylation) or optimization of targeting ligands to balance safety and efficacy.

- Exosome

**Figure 4:**
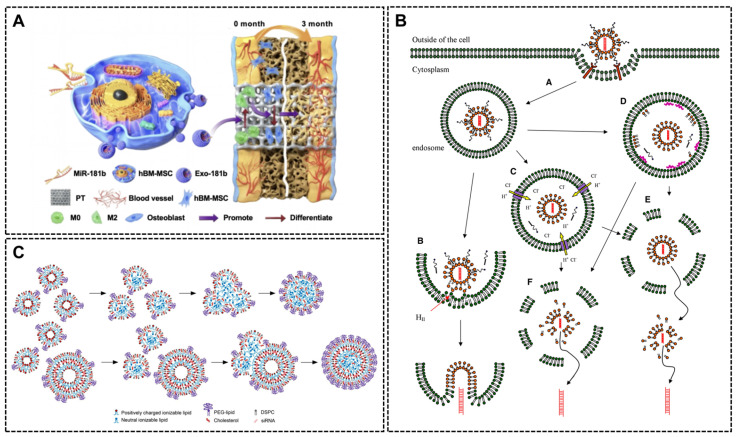
Schematic illustration of the mechanisms of exosomes and lipid-based nucleic acids carriers. (**A**) Schematic illustration for engineered miR-181b exosomes that improved osteointegration by regulating macrophage polarization [51]. Copyright 2021, BioMed Central. (**B**) Proposed mechanisms of formation and structure of LNP prepared in the absence and presence of siRNA [57]. Copyright 2018, American Chemical Society. (**C**) Endosome escape in lipoplex-mediated siRNA delivery [58]. Copyright 2009, Elsevier.

Exosomes are nanoscale vesicles secreted by cells and characterized as low immunogenicity, high biocompatibility and excellent ability to cross biological barriers. They effectively facilitate nucleic acid delivery to targeted cells and promote regeneration through cell-to-cell communication. Nearly all cell types naturally secrete exosomes, including endothelial cells, MSCs, and immune cells [49]. These vesicles can be classified into two groups: natural exosomes and engineered exosome, according to whether they have been artificially modified. Natural exosomes are preloaded with bioactive cargo such as proteins, glycoconjugates, lipids, and nucleic acids [50]. In comparison, engineered exosomes are modified to deliver specific therapeutic molecules as miRNAs and lncRNAs. They can be produced by transfecting targeted genes into stem cells with viral vectors, such as lentivirus and retrovirus [51,52]. This can also be achieved by transfection with non-viral methods like CaCl2 treatment and CD9-HuR fusion protein systems [53,54]. In order to enhance transfection efficiency, especially for difficult-to-transfect cell types like stem cells, advanced electroporation techniques, such as the cellular nanoelectroporation system based on track-etched membranes, have also been developed. These methods ensure cell viability and avoid cytotoxicity concerns while enabling efficient loading of large molecules like CRISPR systems [55]. Electroporation is particularly well suited for CRISPR systems due to its ability to efficiently deliver large macromolecules. By optimizing parameters such as voltage and pulse duration, the loading efficiency of CRISPR components can be significantly enhanced [56]. Once engineered, exosomes deliver nucleic acids cargo into cells via membrane fusion or endocytosis. For example, exosomes loaded with miR-181b were shown to fuse RAW264.7 macrophages, releasing miR-181b which will be overexpressed and regulates the polarization state of macrophages (Figure 4A). Thus, miR-181b promote M2 macrophage polarization and secretion of anti-inflammatory cytokines such as IL-10 to inhibit the inflammatory response by targeting PRKCD and activating the AKT signaling pathway. At the same time, MiR-181b promotes osteogenic factors like VEGF and BMP-2, which ultimately enhance bone regeneration and implant integration with bone tissue indirectly [51].

Despite their promising potential, exosome applications face the following challenges: (1) limited targeting precision without surface modification or engineering strategies; (2) poorly understood drug release mechanisms, complicating spatiotemporal control; (3) their large-scale production faces difficulties in standardization, and the potential immune responses as well as the safety of tumor targeting need to be verified [59].

- Lipid-based systems

Liposomes and lipid nanoparticles (LNPs) have emerged as promising gene delivery platforms, and some have been approved for nucleic acid delivery in clinic due to their low toxicity and no immunogenicity. The difference between liposomes and lipid nanoparticles is mainly in morphology. Structurally, liposomes are lipid bilayer vesicles enclosing a hydrophilic core to embed nucleic acids (Figure 4B), whereas LNPs pack tightly nucleic acids between two lipid monolayers in sandwich formation (Figure 4C) [57].

LNP–nucleic acid complexes can be prepared by microfluidic technology. Lipid components (alcohol phase) and nucleic acid oligomers (aqueous phase) can aggregate in microchannels and self-assemble into stable LNP–nucleic acid complexes [60]. Compared to liposomes, LNPs are thought to be more efficient in siRNA delivery because siRNA and lipid are co-assembled during cellular uptake. LNPs incorporate siRNAs with higher concentrations and significantly enhance siRNA uptake efficiency compared to the liposome delivery system [61]. In addition, LNPs can also be modified by combining with proteins to improve performance. For example, Rabbani et al. introduced supercharged coiled-coil protein (CSP) to the previous cationic lipid nanoparticles (CLNs) to enhance siRNA condensation and improve topical siRNA delivery. Thus, CSP enhances siRNA complexation with a CLN and delivery without compromising liposome elasticity. Moreover, some cationic LNPs have special properties. For example, Yuanfeng Li et al. developed a dual-functional lipid nanocarrier (cLpT@siRNA) that integrates ROS-scavenging lipid LpT (Lipid-pba-Tempo) with cationic lipid DOTAP to condense siMMP9 into ~45 nm stable complexes via electrostatic interactions. The nanocomplexes are internalized by macrophages through macropinocytosis and clathrin-mediated endocytosis, escape endosomes via proton sponge effects, and release siRNA to silence MMP9, while LpT eliminates ROS and polarizes M1 to M2 macrophages. This synergistic modulation alleviates inflammation, preserves the extracellular matrix, and enhances angiogenesis and collagen deposition, leading to significantly accelerated wound closure in diabetic mice [62]. In contrast, liposomes face limitations in systemic delivery of nucleic acids due to reticulo–endothelial system uptake and hepatic clearance [58]. Nevertheless, liposomes remain effective transfection agents due to their straightforward preparation. Commercially available lipid-based reagents are widely used, as they can be directly mixed with nucleic acids to form stable lipoplexes [63,64,65]. In other research, liposomes modified by electrostatic interaction were complexed with pDNA to develop PEGylated SA lipoplexes (pegSA lipoplexes), which increased the residence time of pDNA in the bloodstream and promoted pDNA accumulation in bone marrow, enabling their local delivery and high adhesion [66].

Lipid-based systems, including novel lipidoids and cholesterol derivatives, are versatile non-viral gene delivery platforms for gene therapies in regenerative therapies. However, early cationic liposomes exhibited relatively high toxicity, LNP systems may induce inflammatory responses, necessitating optimization of lipid components. Most studies have focused more on the transfection efficiency of liposomes and LNPs than on their specificity and toxicity [67]. More research is needed in balancing scalability, safety, and adaptability for diverse nucleic acid payloads.

- Peptide-based gene delivery systems

RALA (Ras-like protein Ral-A) is a small GTPase that belongs to the Ras superfamily. Its structure primarily consists of a G-domain and a C-terminal domain. The C-terminal domain of RALA can interact with acidic lipids such as phosphatidylinositol on cell membrane, which helps localize the RALA protein to cell membrane, directing its membrane localization and enabling it to interact with other proteins. Furthermore, RALA contains polar amino acid residues that interact with water (hydrophilic moiety) and non-polar amino acid residues that do not easily interact with water (hydrophobic moiety). This amphipathic fusogenic structure enables RALA self-assembly into nanoparticles (NPs) in aqueous environments and makes it widely recognized for its efficacy in nucleic acid delivery. Chambers et al. firstly demonstrated that RALA can efficiently deliver miR-26a to MSCs in vitro, regulating osteogenic signaling. At an N:P ratio of 6, the RALA/miR-26a complexes can form NPs with a hydrodynamic size of ~74 nm and realize miR-26a complete encapsulation [68]. E. Mulholland et al. developed one RALA with a sequence of “N-WEARLARALARALARHLARALARALRACEA-C” including 11alanines, 10 arginines, and a terminal cysteine residue that facilitates disulfide bond formation or peptide immobilization [69]. This special design protests the cargo associated with RALA from enzymatic degradation, enhanced endosomal escape, and ensures cytoplasmic release [70]. Other modifications such as integrating new fragments, like VGVAPG, can further improve transfection efficiency and serum stability. Research on new designed peptides is also in progress, such as nuclear localization signal peptide (NLS), star-shaped poly(L-lysine) polypeptides (star-PLLs) and cell-penetrating peptide, Golgi-ER transport protein (GET) or heterochromatin protein 1 homolog 1 (Hph-1) [71,72,73]. Nuclear localization signal (NLS) peptides and cell-penetrating peptides (e.g., GET, Hph-1) enable efficient intracellular delivery. Bioinspired star-shaped poly(l-lysine) polypeptides (star-PLL) represent a novel class of non-viral vectors. Advances in N-carboxyanhydride polymerization allow precise synthesis of star-PLLs with controlled molecular weights, the number of attached poly(L-lysine) arms, narrow polydispersity, and tailored architectures while preserving chirality. These polypeptides self-assemble into nano-sized complexes with large quantity [74]. This precision allows researchers to create libraries of star-PLLs with diverse architectural motifs, which can be tailored to match specific cellular uptake preferences. For instance, different cell types may exhibit distinct affinities for specific structural variants of star-PLLs. Additionally, starzPLLs are cost-effective and straightforward to produce. Leveraging their biocompatible peptide side chains, they self-assemble rapidly into nano-sized complexes, capable of encapsulating large nucleic acids payloads. David P. Walsh et al. explored three star-PLL variants and demonstrated their ability to deliver pDNA to MSCs via a rapid clathrin-independent uptake mechanism. This process supports non-taxic transgene expression in vitro [75].

Peptide-based nanomedical platforms hold significant potential for delivering nucleic acids to stem cells in regenerative medicine applications due to their biocompatibility, modular design, and scalable synthesis. Nevertheless, it should be noted that they have similar drawbacks, such as non-specific internalization, fast elimination from the body, intracellular or vesicular entrapment. Several strategies for improving peptide-based gene delivery systems are currently under investigation. The most commonly employed approaches to enhance cellular uptake, selectivity, and stability involve chemical modifications, such as conjugating a stearyl moiety to the N-terminus of acid-activated cell-penetrating peptides or substituting L-amino acids with their D-isomers [67,76].

- Polysaccharide derivatives

Chitosan, a polysaccharide derived from crustacean exoskeletons, possesses positively charged amine groups that enable electrostatic binding to negatively charged nucleic acids like DNA. Chitosan exhibits excellent biocompatibility, biodegradability, and promotes cell adhesion and growth [77]. However, its transfection efficiency has historically been suboptimal, particularly in primary cells. In one study, a 0.1% concentration of 18 KD chitosan nanoparticles achieved a transfection rate of 18.43% compared to 40.57% for commercial lipofectamine (*p* < 0.01) [78]. Transfection efficiency is heavily influenced by molecular weight: high-molecular-weight chitosan is more stable but does not release pDNA efficiently, while low-molecular-weight chitosan enhances pDNA release effectively despite reduced stability [79]. Rosanne M. Raftery et al. investigated two types of chitosan as MSC transfection agents—one is 160 kDa medium-molecular-weight polymeric chitosan, and another is 7.3 kDa low-molecular-weight oligomeric chitosan (OCS). This study has demonstrated that OCS–pDNA nanoparticles at an N/P ratio of 20 carrying 2 μg of pDNA is the most optimal formulation, achieving 45% transfection efficiency in monolayer MSCs without cytotoxicity [80]. Nanoparticle size critically impacts chitosan’s efficacy. Traditional methods like the ionotropic gelation technique or complex coacervation are often employed to yield chitosan–pDNA complexes with polydisperse particles [81,82,83,84]. In contrast, microfluidic systems enable precise control over chitosan nanoparticle formation, producing homogeneous nanoparticles, dense particles with a narrow size distribution and reduced cytotoxicity [85]. By precisely adjusting flow rates, solute concentrations, and channel diameters, researchers can tailor chitosan particle size, shape, and compactness [86].

Hyperbranched polysaccharides, like glycogen and amylopectin, are biocompatible and biodegradable but lack inherent positive charges. To combine with siRNAs, they need to be modified with 1,2-ethylenediamine (EDA) and diethylenetriamine (DETA) to carry positive charges. Biyun Lan et al. modified four types of hyperbranched cationic polysaccharide derivatives (HCP) with glycogen and amylopectin. The HCP/siRNAs complexes safely delivered siRNA via the “proton sponge” effect, facilitating endosomal escape and cytoplasmic release. The result shows that glycogen and amylopectin are both efficient and safe platforms to deliver siRNAs [87]. While their transfection efficiency is influenced by molecular weight and degree of deacetylation, there may be a risk of mild inflammatory responses in vivo. Their utility for other nucleic acids requires further exploration [88].

- Nucleic acid-based nanomaterials

**Figure 5:**
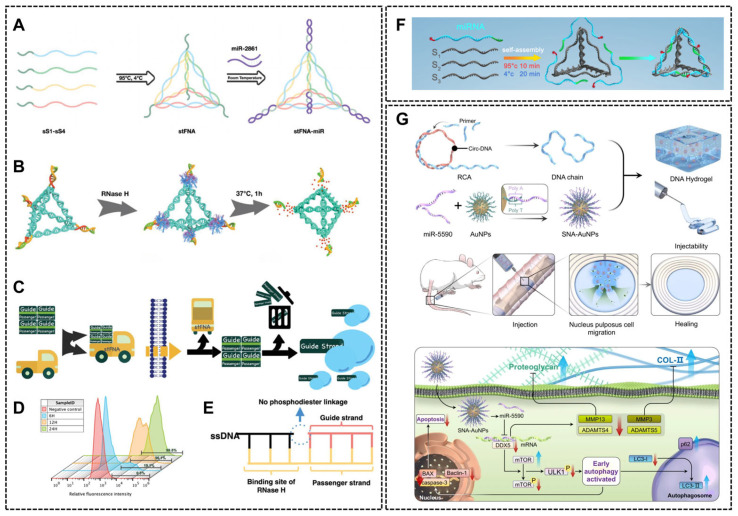
Schematic illustration of the mechanisms of nucleic acid-based nanomaterials. (**A**) Schematic display of the fabrication of stFNA–miR. (**B**) Schematic diagram of the enzyme cleavage test in an extracellular environment. (**C**) Schematic illustration of miRNA transportation into cells by stFNA and selection of a guide strand to realize subsequent biochemical reactions. (**D**) Flow cytometric results for cellular uptake of stFNA. (**E**) The binding site of RNase H and the position of the guide and passenger strands [93]. Copyright 2021, Wiley-VCH. (**F**) The inner core of the BiRDS and the location of the surrounding miRNAs [94]. Copyright 2024, American Chemical Society. (**G**) The procedure used to prepare miR-5590-SNA@DNAgel [97]. Copyright 2023, BioMed Central.

Nucleic acid-based nanomaterials have emerged as versatile platforms due to their precise structural programmability. Tetrahedral framework nucleic acids (tFNAs) are pyramid-shaped, three-dimensional nanostructures assembled via complementary base pairing of four single-stranded DNA. They exhibit straightforward synthesis, wide applicability, structural stability, self-assembly capability, antioxidant properties and anti-inflammatory effects [89,90]. tFNA are categorized into three generations, with the third generation being the most widely utilized. These structure-controlled tFNA nanoparticles undergo a multi-step assembly in response to external stimuli to achieve precise drug release [91]. For instance, four copies of miRNA-29c can be attached to the four vertices of tFNAs via complementary base pairing. In vivo, tFNA enhanced miR-29c delivery and cell membrane penetration, and fully repaired a critical-size skull defect in murine models after 2 months of administration. In vitro experiments showed that stFNA-miR-29c could promote osteogenic differentiation in BMSCs, suppress lipogenesis, and improve cell viability [92]. Also, each vertex of tFNA allows modification of multiple miRNAs at distinct vertices, enabling synergistic regulation of shared biochemical pathways. For instance, S.H. Li et al. modified the 5′ ends of four single-stranded DNAs with sticky ends to obtain s1-4tFNA with four sticky-end apexes, and each sticky end can be hyped with one miR-2661 by DNA/RNA hybridization (Figure 5A). RNase H degrades RNA in RNA/DNA hybrid molecules, only slightly affecting the single- or double-stranded RNA molecules (Figure 5B,E). The stFNA is like a “truck” that transports miRNAs into cells and selects a guide strand to produce subsequent biochemical reactions (Figure 5C). The flow cytometry data showed that the BMSCs had adsorbed a large amount of stFNA during 12 h (Figure 5D) [93]. However, conventionally tFNAs allow miRNAs to bind to either their apical or sidewall, this structure do not adequately protect miRNAs in a complex serum environment. To address this, Xiaoying Lyu et al. developed a tFNA-based bioswitchable miRNA delivery system (BiRDS), where three identical miRNAs are spatially confined to one face of tetrahedron. This design preserves the tFNA’s tetrahedral structure while improving miRNA stability and delivery efficiency (Figure 5F) [94]. tFNAs exhibit intrinsic cell-penetrating capabilities, bypassing the need for any transfection agents to achieve high nucleic acid delivery efficiency [95,96]. Another class of nucleic acid nanomaterials, spherical nucleic acid (SNA)-based vectors, are usually gold nanoparticles in the core and densely loaded with oligonucleotides on the surface. These surface-bound oligonucleotides enable targeted binding and delivery of nucleic acid drugs (Figure 5G) [97].

Despite their promise, nucleic acid vectors face biosafety challenges, including immunogenicity and off-target effects, which require rigorous evaluation for clinical translation. Furthermore, the potential toxicity of long-term in vivo degradation products (e.g., oligonucleotides) requires evaluation, and the cost remains relatively high.

### 4.3. Innovations and Optimization of Synthetic Vectors

#### 4.3.1. Polymer Used for Nucleic Acid Delivery Systems

- Cationic polymers

Polyethylenimine (PEI), a cationic polymer that binds electrostatically to negatively charged nucleic acids, is most frequently used in nucleic acids modification. PEI enhances cellular uptake and gene silencing efficiency [98,99]. Although PEI exhibits dose-dependent cytotoxicity, this can be mitigated by grafting negatively charged phenylboronic acid (PBA) groups onto its branched chains or by modifying it with bile acids [100,101]. Branched polyethylenimine (bPEI) variants reduce cytotoxicity while maintaining high transfection efficiency and enabling homogeneous pDNA encapsulation into spherical nanoparticles [102,103]. Meanwhile, the combination of PEI with polyethylene glycol (PEG) reduces its cytotoxicity by decreasing the positive charge of PEI [104,105]. Other cationic polymers, including polybutyl cyanoacrylate (PBCA), poly(β-aminoester) (PBAE), and polylactic acid–glycolic acid copolymer grafted polyethylene (PgP) serve a role similar to that of PEI [106,107,108]. These hydrophilic polymers are often co-used to form core–shell structures that protect nucleic acid stabilization in the core and regulate the release of nucleic acids by adjusting the permeability of the shell [109]. Polylactic acid–glycolic acid copolymer (PLGA) nanoparticles, often prepared via the double-emulsion method, can be surface modified to target specific cells. For instance, modifying its surface with galactose (Gal) effectively enhances PLGA-nanoparticle’s capability to target macrophages [110,111]. PEI-modification of PLGA can enhance its connections with nucleic acid drugs [112].

The high positive charge density of cationic polymers leads to cell membrane disruption, increased lysosomal membrane permeability, called the “proton sponge effect” of PEI, and activation of the mitochondrial apoptotic pathway. Some ligands, such as arginine-modified ones, enhance delivery efficiency but may increase the risk of immunogenicity. To address this limitation, regulating surface charge emerges as a central strategy for attenuating the in vivo toxicity of cationic polymers. By neutralizing the carrier’s positive surface charge, electrostatic interactions with cell membranes are diminished, markedly reducing membrane disruption and cytotoxicity [113].

- Polyamide amine dendrimers

Polyamide amine (PAMAM) dendrimers, with a highly branched 3D structure, form stable complexes with nucleic acids to enhance intracellular uptake and gene silencing efficiency. Amino-terminated fifth-generation (G5) PAMAMs are commonly used due to their capability to bind pDNA and high affinity for MSCs surface receptor [114,115,116]. To reduce cytotoxicity, PAMAMs are modified with basic amino acids such as histidine, lysine, and arginine. These PAMAM derivatives enhance gene transmission by increasing cell uptake and endoplasmic reticulum escape capacity. These PAMAM derivatives increase endosomal pH via the proton sponge effect, which triggers the endoplasmic reticulum rupture and facilitates the release of pDNA into the cytoplasm [117]. Ganjun Feng et al. have shown hyperbranched polymer (HP), where PEG chain and PEI are attached to the hydrophobic cores, exhibits high pDNA binding affinity and negligible cytotoxicity [118]. Nucleic acid-polymer complexes can also self-assemble into stimuli-responsive micelles, which are capable of changing their structure and performance or achieving controlled release in response to the external environment. For instance, polyplex micelles are formed through the self-assembly of RGD peptide-conjugated with poly(ethylene glycol)-polylysine (PEG-PLys) and pDNA [119]. Alternatively, pDNA can be combined with mixed cationic block copolymers, such as PEG (polyethylene glycol)-b-poly aspartic acid and PNIPAM (poly(N-isopropylacrylamide))-b-poly aspartic acid, at 25 °C. The DET moiety, containing amino groups, facilitates electrostatic interactions with pDNA’s phosphate backbone. These micelles undergo structural reorganization, forming a heterogeneous surface layer composed of hydrophobic and hydrophilic microdomains when heated to 37 °C. This composes thermosensitive polycomposite micelles, which enhance nuclease resistance and protest against nuclease degradation in vivo. Additionally, the optimized surface charge distribution of the micelles reduces non-specific interactions with proteins, further enhancing their biocompatibility and functional efficacy [120].

Notably, inorganic polymers can be designed in both nanoparticles and nanomicelles and further modified through conjugation with diverse molecules. Inorganic nanoparticles used for gene delivery are usually biocompatible. However, their toxic properties can change depending on size and surface coating. It was shown that poor biodegradability and the lack of an excretion mechanism in high-molecular-weight PEI leads to significant cytotoxicity in vitro. A few years after the occurrence, the hypothesis of necrotic cell death during PEI polyplex internalization was proven. The results of the cytotoxicity mechanism study showed that cell membrane destabilization occurs with some cationic polyplex interactions, such as PEI and PLL, in a dose-dependent manner [121]. Currently, optimization of polymer structure, regulation of surface charge, targeted delivery, and development of degradable materials represent core strategies to reduce the in vivo toxicity of cationic polymers. For polymer structure optimization, the design of degradable backbones can be employed: polymers containing cleavable bonds (e.g., ester bonds), such as poly(β-amino ester) (PBAE) and poly(amino co-ester) (PACE), can degrade in vivo and reduce long-term toxicity. PACE polymers reduce systemic toxicity through ester bond hydrolysis while maintaining efficient transfection. Surface charge regulation can reduce electrostatic interactions with cell membranes by neutralizing the positive charge on the carrier surface, thereby significantly mitigating membrane damage and lowering cytotoxicity. Targeted delivery enhances the specificity of carriers for diseased tissues via conjugation with targeting ligands (e.g., RGD peptides, folic acid, and antibodies), which reduces non-targeted accumulation and systemic exposure, consequently decreasing the effective dose and toxicity. The development of novel degradable materials, such as polyether dendrimers and phosphorous-containing dendrimers, minimizes long-term toxicity through their degradable backbones while retaining efficient nucleic acid loading capacity [122]. Their huge potentials in biomedical application warrants further investigation to fully exploit their capabilities.

#### 4.3.2. Inorganic Salt-Based Nanomaterials

Mesoporous silica nanoparticles possess hundreds of empty, tunable mesopores capable of encapsulating diverse bioactive molecules, including nucleic acids. The size, volume and structure of these pores can be adjusted according to the size and properties of the cargo dimensions and properties [123,124]. MSNs exhibit low cytotoxicity due to their gradual degradation into orthosilicic acid (Si(OH)_4_), which is easily excreted via the kidneys. These properties make them promising candidates for drug delivery. For instance, Radu et al. demonstrated that G2-PAMAM-capped MSNs protect pDNA from enzymatic cleavage while enabling gene expression in HeLa cells [125]. MSN–nucleic acid complexes often exhibit anti-inflammatory properties, and even empty MSNs have also shown anti-inflammatory potential by inhibiting the activation of the NF-κB signaling pathway via TLR2 downregulation. MSNs loaded miR-21-5p promotes angiogenesis and mature vessel formation by targeting SPRY1 and subsequently activating VEGF-induced ERK-MAPK signaling [126]. Yan Li et al. engineered MSNs with amino and trimethylamines to positively charge their surfaces, which stabilizes MSN interaction with negatively charged miRNAs [123]. Stimuli-responsive MSNs, such as disulfide bonded MSN-S-S-NH2, allow MSNs to release loaded miR222 intracellularly by a redox reaction with endogenous GSH [127]. Beyond MSNs, other mesoporous carriers, including magnesium silicate nanospheres with PEI-enhanced oligonucleotide loading capacity [128] and dual-pore structure calcium–silicon nanospheres (DPNPs), are biocompatible, morphology controllable, and bioactive. They can co-encapsulate miRNA-210 and simvastatin (Siv) and significantly accelerate the bone repair process in vitro and in vivo [129].

Gold nanoparticles (GNPs) are another versatile platform for nucleic acid delivery. Biosynthesized using Lignosus rhinocerotis extracts, GNPs exhibit antimicrobial properties [130]. Nucleic acid drugs usually bind to GNPs modified with positive and negative cationic polymers, like PEI, polylysine and PEG-SH (polyethylene glycol-SH). These modifications improve GNPs’ stability, reduce the immune response and minimize non-specific protein interaction [131,132,133]. Similarly, cerium oxide nanoparticles (CNPs) have been widely used in the treatment of oxidative stress-related diseases [134]. Because of oxygen defects in their lattice structures, CNPs can also act as a regenerative free radical scavenger by the redox cycle between the Ce3+ and Ce4+ oxidation states [135], which reduce all harmful intracellular reactive oxygen species (ROS), mimicking antioxidant enzymes like superoxide dismutase and catalase [136]. When conjugated with miRNAs, CNP–miRNA complexes synergistically target both oxidative stress and inflammation [137]. Also, functionalized graphene oxide (GO), surface modified with PEG and PE, enabled efficient miRNA encapsulation via electrostatic interaction and π–π interactions, greatly improving their biocompatibility and anti-inflammatory properties [138].

While inorganic nanomaterials offer immense potential due to their structural and functional plasticity, as well as their tunable physicochemical properties, including size, surface charge, and stimuli responsiveness, their long-term biosafety remains a critical consideration. The long-term bioaccumulation of silica-based nanoparticles, MSNs and CNPs poses significant risks to chronic tissue remodeling, primarily through persistent inflammation, fibrotic progression, and disruption of redox homeostasis. It is reported that accumulation of non-degradable silica in the liver and spleen triggers chronic macrophage activation, which in turn leads to excessive collagen deposition and fibrotic scarring. Long-term studies show that persistent silica residues induce granulomatous lesions, disrupting normal tissue architecture [139]. While CNPs exhibit antioxidant activity through Ce^3+^/Ce^4+^ redox cycling, their long-term accumulation can paradoxically induce pro-oxidant effects in specific microenvironments, especially acidic lysosomes. This disruption of cellular redox signaling causes damage to lipids and DNA, and activates senescence pathways. Additionally, nanoparticle aggregates can obstruct renal tubules, reducing filtration capacity and promoting tubular atrophy [140]. Mitigation strategies for these risks may involve the design of rapidly biodegradable MSNs that transform into renally clearable silicic acid, thereby minimizing residual accumulation. For CNPs, surface modification with dextran or PEG has been shown to enhance renal clearance, with 99% of CNPs cleared within 72 h in murine models. Moreover, optimizing CNPs to sizes below 5 nm improves renal excretion and reduces hepatic and splenic sequestration. Similarly, MSNs with enlarged mesopores (>10 nm) facilitate more rapid biodegradation [141]. Bioaccumulation of inorganic nanomaterials poses multifaceted risks to tissue remodeling, necessitating material innovation, such as biodegradable scaffolds, and dosing precision. Future work must prioritize longitudinal toxicity studies and clinically relevant clearance strategies to bridge translational gaps.

## 5. Application of in Site Delivery Systems in Gene Therapy

In situ delivery systems hold significant promise for advancing gene therapy by using three-dimensional (3D) cellular environments, which offer critical advantages over conventional two-dimensional (2D) cultures. Unlike 2D systems, 3D systems provide the 3D spatial structure and dynamic mechanical stimulation (e.g., fluid shear stress) essential for physiological cell growth and function [142]. Furthermore, 3D environments enhance cellular differentiation by enabling self-assemble to form spheroids or organoid structures, with the extracellular matrix (ECM) composition and organization similar to native tissues [143]. In situ tissue engineering scaffolds exemplify this approach by providing a biomimetic 3D environment. They not only serve as carriers for nucleic acid delivery but also recruit endogenous stem cells migration to the site, facilitating their proliferation and differentiation. These scaffolds are categorized into two main types based on their application and morphology: in situ injectable scaffolds and 3D scaffolds.

### 5.1. An Injectable Delivery System for Nucleic Acid Therapy

Injectable scaffolds enable minimally invasive nucleic acid delivery to damaged tissues via a syringe or catheter injection. Typically composed of hydrogels, these systems exhibit shear-thinning property—reduced viscosity under high shear stress—which allows smooth injection under applied force [144]. After injection, the hydrogels undergo rapid gelation in situ, forming stable gel structures and fill the irregularly shaped cavities. This localized delivery mechanism improves nucleic acid concentration at target sites while minimizing “off-target” distribution, thereby reducing systemic side effects and improving therapeutic precision [145,146,147]. Key designs and applications of injectable delivery systems are summarized in Table 1.

#### 5.1.1. Design for Sustained and Sequential Release

Hydrogels enable sustained and sequential nucleic acid release through mechanisms such as degradation, swelling, and diffusion. As the hydrogel degrades, the mesh size expands, allowing nucleic acids encapsulated to diffuse out. Swelling and diffusion. Swelling-induced structure changes in the hydrogel further modulate the release kinetics. Tailoring hydrogel properties, including concentration, physical architecture, the cross-linking method, and cross-linking density, allows precise control over nucleic acids sustained and sequential release [137,152,178]. For instance, Laponite nanoparticles are a kind of synthetic Hectorite with a disc-like structure approximately 25 nanometers in diameter and 1 nanometer thick and charge heterogeneity, which negatively charged surfaces and positively charged edges. These nanoparticles form reversible microporous gel networks, which permit slowly releasing bioactive molecules like miR-22 via face-edge interactions [170]. Similarly, Wang et al. synthesized photopolymerized hydrogels by combining PEG and PLA chains modified by methacrylate (DM) groups. This PEG-PLA-DM hydrogel network gradually reduces cross-linking density by cleavage of PLA ester bonds, loosens the network structure, and accelerates nucleic acid release over time [157]. Release rates are also influenced by nucleic acid-scaffold binding modes, such as electrostatic adsorption, covalent conjugation, and hydrophobic interaction.

Despite these advances, few novel designs are available for sustained release systems. Further research is necessary to optimizer the physical and chemical properties of hydrogels, such as swelling dynamics and degradation rates. More importantly, tissue- and cell-specific release kinetics need to be refined, like how varying release rates impact therapeutic efficacy and potential side effects across diverse cellular environments.

#### 5.1.2. Designs for Stimuli-Responsive Systems

Localized rejecting systems enable site-specific nucleic acid administration, enhancing tissue targeting while minimizing off-target effects. These systems exhibit strong bio-adhesion, allowing conformal coverage of irregular wound surfaces. The advantages can be further enhanced by in situ formed hydrogels, including temperature-sensitive hydrogels and light-curing hydrogels, further optimizing these advantages by leveraging environmental stimuli to achieve precise spatial–temporal control over gelation and drug release.

- Photosensitive hydrogel

**Figure 6:**
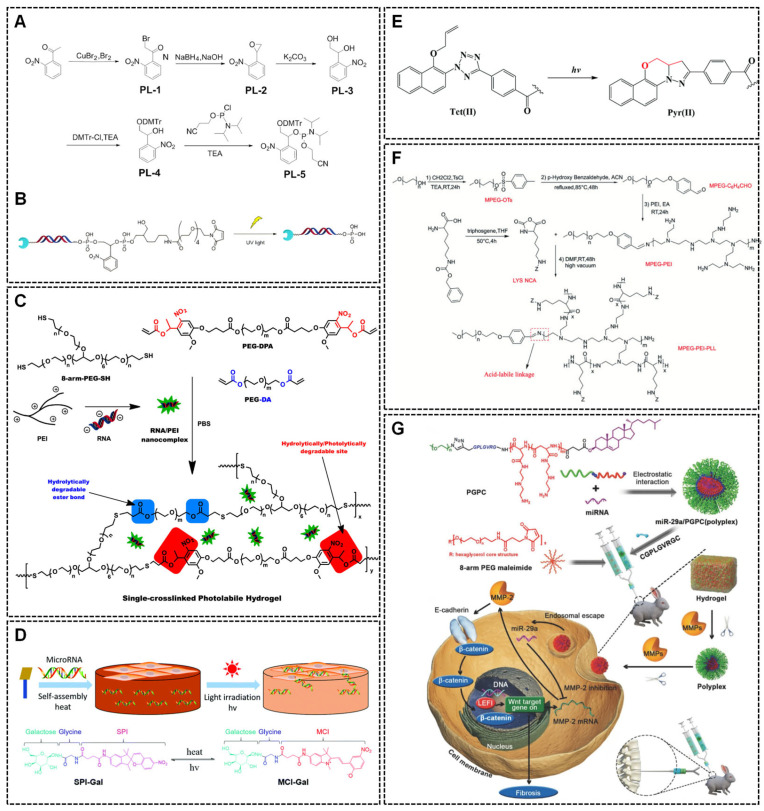
Designs for stimuli-responsive release and chemical strategies. (**A**) Synthesis of the photocleavable linker (PL). (**B**) Chemical strategies for miR-26a conjugation with a photocleavable linker (PL-5) and photosensitivity mechanism [148]. Copyright 2021, Elsevier. (**C**) Formation of RNA/PEI nanocomplexes and hydrogel fabrication via a single cross-linked Michael addition reaction [181]. Copyright 2017, American Chemical Society. (**D**) Dual-functional supramolecular hydrogel design for targeted and light-responsive miRNA delivery, including structure transformation between SPI–Gal and MCI–Gal under heat or light control [185]. Copyright 2016, Royal Society of Chemistry. (**E**) Chemical structure and intra-molecular photo-click reaction of Tet (II) [186]. Copyright 2017, Royal Society of Chemistry. (**F**) Synthesis of MPEG-PEI-PBLL [187]. Copyright 2020, Royal Society of Chemistry. (**G**) Formation of miRNA/PGPC polyplex micelles, encapsulation in injectable PEG hydrogels, and molecular mechanism of MMP-2 silencing in nucleus pulposus cells for fibrosis inhibition [150]. Copyright 2018, Wiley-VCH.

Photosensitive hydrogels can be classified into photocleavable and photopolymerizable types. Photocleavable hydrogels employ light of a specific wavelength to trigger nucleic acid release; their scaffold materials often incorporate photosensitive moieties, such as o-nitrobenzyl groups or photodegradable cross-linkers. Photopolymerizable hydrogels, on the other hand, undergo a light-induced polymerization of photosensitive components to form a 3D network structure. Photolyzed hydrogel is a highly controllable platform for nucleic acid delivery, leveraging specific wavelength of light to nucleic acid release. These systems are often fabricated using light-responsive hydrogel scaffolds synthesized via Michael addition reactions. Scaffold materials often contain photosensitive groups, such as o-nitrophenyl structure or photodegradable cross-linkers. For instance, Minfeng Gan et al. prepared a photosensitive moiety containing the o-nitrophenyl group to realize the controlled release of miR-26a on demand (Figure 6A). The 3′ end of miRNA-26a was conjugated with the photolytic group (PL-5) containing an o-nitrophenyl structure, enabling ultraviolet (UV)-triggered cleavage and miRNA release (Figure 6B) [148]. Poly(ethylene glycol)-diacrylate (PEG-DA) and PEG-diphotodegradable-acrylate (PEG-DPA) macromers are common hydrogels cross-linked via thiolene click reactions with 8-arm PEG mercaptan (PEG(-SH)8) to form photolabile thiol-acrylate networks (Figure 6C) [181,182,183]. Upon absorption of UV light, the chemical bond undergoes Norrish type I or type II cleavage, and the ester group breaks down into carboxylic acid and alcohol which in turn promotes the degradation of the hydrogel [184]. Notably, the release of nucleic acid is not exclusively light-sensitive hydrogels. siRNAs exhibit baseline diffusion without illumination, while UV accelerates gel degradation [182]. Alternative strategies utilize chemical compound, such as biaryltetrazole (Tet) and or spiropyran (SP) derivatives [185,186]. Tet (II) modified with RGD peptides form hydrogels in PBS at neutral pH, undergoing rapid intra-molecular photo-click reactions under UV light (302 nm) to convert Tet (II) into Tet (II)–GRGD, Tet (II)–GGRGD and Tet (II)–GGGRGD (Figure 6E). Then, Tet (II)–GRGDS transform completely into Pyr(II)–GRGDS within 10 min [186]. Similarly, the photoisomerized spiropyran-galactose (SP-Gal) was synthesized and assembled by linking SP to Gal via a strong π–π stacking of merocyanine (MC) groups upon heating. Visible light irradiation triggers MC-to-SP isomerization, disrupting the hydrogel network, transform the hydrogel into solution and releasing miRNAs (Figure 6D) [185].

While photolyzed hydrogels show promise, most studies remain confined to in vitro models. Critical gaps persist in understanding photodegradation kinetics, biocompatibility, and therapeutic efficacy in vivo. Optimizing light penetration depth, minimizing UV-induced tissue damage, and tailoring wavelength-specific systems for clinical translation are key priorities.

**Figure 7:**
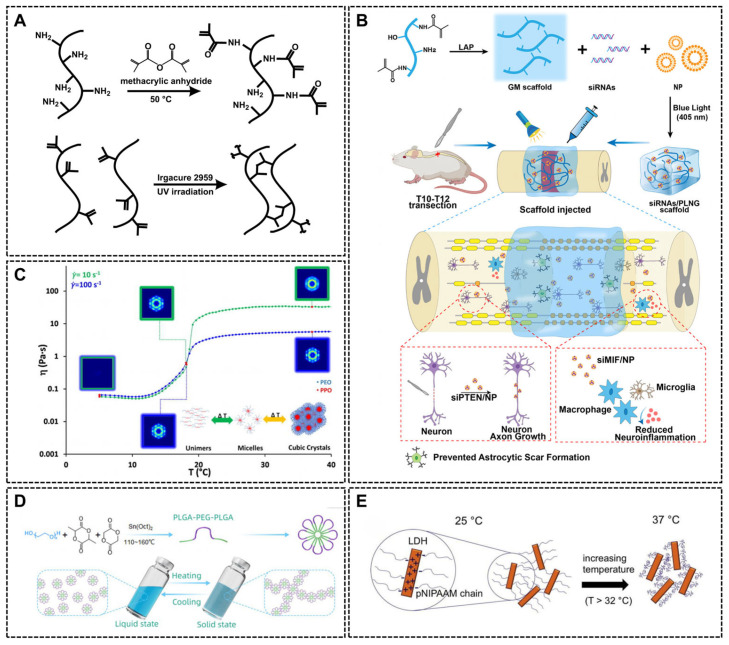
Photosensitive and temperature-sensitive hydrogels for biomedical applications. (**A**) Synthesis of methacrylated gelatin [188]. Copyright 2010, Elsevier. (**B**) The synthesis and therapeutic effect of the siRNAs/PLNG scaffold for the treatment of SCI [155]. Copyright 2024, American Chemical Society. (**C**) The structural changes in F127 systems under controlled temperature after flow [190]. Copyright 2020, Elsevier. (**D**) The synthesis and mechanism of action of the heat-sensitive hydrogel be made up of PLGA–PEG–PLGA [102]. Copyright 2023, AIP publishing. (**E**) An illustration of how the polymer solution transits from a soluble state to a viscous hydrogel by hydrophobic interactions between the isopropyl groups of pNIPAAM, when the temperature is above its lower critical solution temperature (LCST), 32 °C [191]. Copyright 2015, Elsevier.

Another type of light-responsive hydrogel is photopolymerized hydrogel. Over the past few years, as a kind of photo-cross-linked biological hydrogel, gelatin methacryloyl (GelMA) hydrogel has emerged as a leading photo-cross-linked biomaterial. Synthesized by adding methacrylate groups to amine residues in gelatin, GelMA incorporates photosensitive groups enabling precise photopolymerization. When exposed to light, the photoinitiator decomposes and releases free radicals, which initiate methacrylamide cross-linking network and eventually form a tunable three-dimensional GelMA (Figure 7A) [188]. This light-responsive gelatin allows precisely spatially controlled nucleic acid delivery to the complex defect site, such as skin wounds and spinal cord injury (Figure 7B) [128,152,155]. The photopolymerization strategy can be applied extensively to chitosan via a nucleophilic substitution reaction. The hydroxyl groups on chitosan react with compounds containing double bonds, such as glycerol methacrylate and GelMA, introducing formyl groups [156]. Riboflavin, a kind of biocompatible photoinitiator, generates radicals when exposed to blue light to cross-link methacrylated polymers like methacrylated glycol chitosan (MeGC) [156,189]. This methodology is adaptable to some other synthetic polymers such as PEG-DA [134,157].

Despite their utility, photopolymerized hydrogels face three key limitations. Firstly, cytotoxicity risks, photoinitiators, like riboflavin, generate free radicals that may leave cytotoxic residues if incompletely consumed during polymerization. More research needs to be applied in developing enzyme-initiated polymerization or metal-free photoinitiators. Secondly, they have limited chemical diversity. Current systems rely mainly on methacrylate groups, with underutilized alternatives like acrylamide or thiol-ene chemistries restricting functional optimization. Exploring norbornene–acrylate hybrids and tetrazole-alkene click chemistry maybe a solution for the development of the next-generation photosensitive system. Thirdly, light penetration remains the principal bottleneck to clinical translation. Clinically, the near-infrared range, UV and blue light, remains limited tissue penetration depths (<2 mm), restricting current photo-responsive hydrogels to superficial sites like skin and cornea or lesions reachable by minimally invasive tools as endoscopically accessible tumors. Raising power to compensate risks collateral photodamage without adequately treating deeper tissue.

To overcome this, four future strategies are emerging: (1) technical synergy—pairing hydrogels with optical fibers, micro-LED implants, or catheter-based endoscopes that deliver light directly to the lesion; (2) alternative stimuli—using more penetrative triggers such as focused ultrasound, magnetic hyperthermia, or low-dose X-ray to indirectly activate photosensitizers or photothermal converters, thereby achieving “remote” light generation in situ; (3) material innovations—engineering ultra-sensitive photosensitizers activatable at very low light fluence and smart carriers that persist at the lesion site and respond to weak local optical signals; and (4) targeted accumulation—enhancing hydrogel density within deep lesions through active targeting (e.g., antibody, peptide, or metabolic ligands) so that therapeutic responses occur under strictly confined, low-intensity illumination.

- pH-responsive hydrogels

PH-responsive hydrogels typically exploit dynamic Schiff-base bonds formed between amines and active carbonyl groups. These bonds remain relatively stable under neutral or alkaline conditions but rapidly degrade under acidic environments, enabling targeted delivery and controlled drug release at specific pH values. This property allows targeting delivery via Schiff-base bond breaking at a specific pH value, thus achieving a controlled drug release [192]. For instance, in pathological tissues like tumor where pH value is approximately 5.0, Schiff-base bond-based hydrogels enable targeted drug release (Figure 6F) [187]. In tissue engineering, Yan Li et al. developed an injectable hydrogel (Gel@MSN/miR-21-5p) by cross-linking amino-modified MSN (MSN-NH2-TMA) with aldehyde-modified polyethylene glycol (PEGCHO) and cyclodextrin (α-CD). The Schiff base ensures stability at physiological pH 7.4 but gradually degrades in an acidic environment (pH 6.8), enabling localized miRNA-21-5p release to promote angiogenesis in myocardial infarction [126]. In conclusion, pH-responsive hydrogels are superior for tissue targeting.

- Enzyme-responsive hydrogels

Enzyme-responsive hydrogels exploit proteases that are upregulated in injured tissues. For instance, matrix metalloproteinases (MMPs) are highly expressed in osteoarthritis and intervertebral disc degeneration. By incorporating MMP-cleavable peptide sequences into the hydrogel network, the scaffold selectively degrades in response to elevated MMP activity, enabling a stage-wise delivery system. A research shows that the peptide linkage GPLGVRG can be cleaved specifically by a series of MMPs [193]. Building on this approach, the MMP-cleavable peptide CGPLGVRGC was integrated with eight-arm PEG–maleimide solution to enable sustained release of miRNA-29 from MMP-responsive copolymers carriers (Figure 6G). This system achieves a two-stage release: In the first stage, elevated MMP levels trigger the degradation of the hydrogel and release polycomposite micelles. In the second stage, polycomposite micelles undergo MMP-triggered breakdown and facilitate intracellular miRNA uptake and their endoplasmic reticulum escape. When combined into a hybrid hydrogel (miR-29a/PGPC@HG), this strategy significantly attenuated fibrosis in intervertebral disc (IVD) degeneration by targeting miR-29a to diseased tissue [150]. Similarly, hyaluronidase-responsive systems exploit enzyme cleavage of β-N-acetylhexosamine-1,4 glycosidic bonds in hyaluronic acid (HA). An injectable hybrid hydrogel synthesized via the Michael addition reaction of hyperbranched poly(ethylene glycol) diacrylate (HB-PEGDA) and thiolated HA (SH-HA) degrades in osteoarthritic joints, where hyaluronidase and H_2_O_2_ synergistically trigger sustained drug release from HA-based microparticles [151]. Emerging designs also incorporate dynamic hydrazine bonds and protease-sensitive peptide cross-linkers [145]. Enzyme-cleavable hydrogels rely on the assumption that the target protease is both present and active at the intended site. Their performance, especially those triggered by matrix metalloproteinases, hinges on the actual abundance, activity, and spatial distribution of the target enzyme within the lesion. However, three layers of heterogeneity can compromise this assumption. First, inter-patient variability, like genetic polymorphisms, comorbidities, diet, and microbiome differences, may create patient to patient variation in protease baseline and enzyme expression. Second, enzyme levels rise and fall across disease stages, which may result in transiently overshoot in acute flares, whereas downregulation or compensatory inhibition in chronic lesions. Third, intra-lesion heterogeneity, like spatial gradients in tumor core and invasive rim, produces micro-zones where enzyme abundance differs. These heterogeneities of proteins and enzymes in vivo may result in off-target activation in adjacent healthy tissues with aberrant enzyme expression or negative release when enzyme concentration falls below the activation threshold.

To overcome these challenges, next-generation enzyme-responsive hydrogels can be engineered through three complementary strategies: (1) incorporating cleavage sites for multiple disease-associated enzymes to heighten specificity and mitigate single-marker failures; (2) tuning the activation thresholds of these cutting sites to accommodate the wide range of enzyme activities observed across patients and disease stages; and (3) integrating additional microenvironmental triggers, such as acidic pH, elevated ROS, or altered redox states, to ensure robust activation even when enzyme levels fluctuate. Together, these design principles promise safer and more reliable hydrogels for clinical translation.

- Temperature-sensitive hydrogel

Temperature-sensitive hydrogels undergo reversible sol- and gel-phase transition at physiological temperatures, which enable minimal invasive injection and enhance the localized adhesion. For instance, PEG-PLGA-PNIPAM increases the viscosity to form a hydrogel at temperatures above 33 °C and returns to a low-viscosity solution state at room temperature (25 °C). ASP and miR222/MSN can be encapsulated in PEG-PLGA-PNIPAM without structural compromise and retain their spherical structure [127]. The pluronic F-127 (PF-127) hydrogel, an FDA (Food Drug Administration) cleared thermosensitive polymer for clinical use, is a triblock copolymer containing one hydrophobic polypropylene oxide (PPO) chain and two hydrophilic polyethylene oxide (PEO) chains. At lower temperatures, PF-127 molecule tends to form micelles in which hydrophobic PPO chains gather inside the micelles and hydrophilic PEO chains extend outward. At 37 °C, these micelles re-arrange into large-scale organized structures, forming a hydrogel encapsulating shRNA-lentiviral complexes and releasing them persistently (Figure 7C) [20,164,190]. Another triblock copolymer, PLGA–PEG–PLGA, is micelles at low temperature and re-arranges into woven networks at 37 °C via hydrophobic interactions, enabling controlled nucleic acid release (Figure 7D). The nanomicelles formed by this copolymer in water change from a random distribution to a regular linear arrangement at an elevated temperature (37 °C), forming an interwoven network of micelles, which ultimately leads to the curing of the hydrogel. The phase transition caused by this temperature change is due to the fact that the PEG chains are more stretched at lower temperatures and more coiled due to hydration at higher temperatures, which promotes the interaction between the polymer chains and forms a stable hydrogel network, thus realizing the local delivery and controlled release of nucleic acid drugs [102,163].

The cross-linking can be modulated by natural or synthetic hybrids. Hydrogels prepared by mixing chitosan and β-glycerophosphate for delivery of pDNA remain liquid at room temperature and rapidly transform to gelation at b 37 °C by ionic cross-linking between chitosan and β-GP, as well as chitosan chain entanglement, which protecting pDNA structure [81,84]. Similarly, pNIPAAM solution changes from a soluble state to a gelation due to enhanced hydrophobic interactions between pNIPAAM molecules (mainly isopropyl-based interactions) at 32 °C. In this system, siRNAs are loaded on positively charged layered silicates, which forms stable ion exchange with sulfonate groups in pNIPAAM, stabilizing siRNA-loaded hydrogel networks (Figure 7E) [191]. pNIPAAM copolymerized with Dex-PCL-HEMA improves biocompatibility and biofunctionality of the complex [165].

These designs face critical challenges such as strict temperature control because of premature gelation in vivo and thermal instability. Even 1 or 2 °C temperature fluctuation may trigger burst release of nucleic acids and structural collapse.

#### 5.1.3. Design for In Situ Self-Assemble Hydrogels

Unlike stimuli-forming hydrogels, self-assembling hydrogels form stable, ordered structures in situ without external triggers. They leverage intrinsic molecular recognition to spontaneously organize at target sites, maximizing therapeutic precision. These systems comprise two primary classes: the host–guest self-assembly and the peptide self-assembly.

- Host–guest self-assembling hydrogels

**Figure 8:**
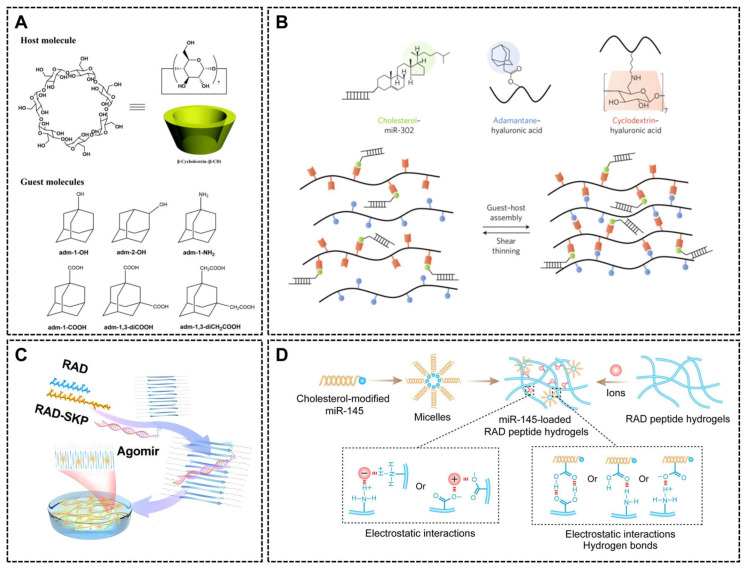
Designs of injectable hydrogels for in situ self-assemble and localized release. **(A)** Chemical structures and cartoon illustration of β-CD and the adamantane derivatives [194]. Copyright 2021, MDPI AG. Licensed under CC BY 4.0. (**B**) Hyaluronic acid modified by Adamantane and cyclodextrin lead to hydrogel self-assembly via guest–host interactions, and disassembly on shear thinning. MiR-302 modified by cholesterol allows its incorporation into the gel via the guest–host interaction of cholesterol with cyclodextrin [166]. Copyright 2017, Nature Research. (**C**) Schematic illustration of SKP@miR [167]. Copyright 2022, American Association for the Advancement of Science. Licensed under CC BY-NC 4.0. (**D**) Fabrication and gelation mechanisms of miR-145-loaded RAD peptide hydrogels [168]. Copyright 2024, Elsevier.

The host–guest hydrogels leverage molecular recognition between β-cyclodextrins (β-CDs) hosts and hydrophobic guests such as adamantane (AD) to form injectable, self-assembling networks. β-CD crystallizes as a pair supported by face-to-face hydrogen bonding and form truncated-cone-shaped cavities stabilized by hydroxyl hydrogen bonding on C2 and C3 (Figure 8A) [194]. The β-CD (host) and AD (guest) self-assemble via hydrophobic interactions to form a gel exhibiting shear-thinning properties during injection, and rapidly self-healing after removal of the shear [144]. Meanwhile, the hydrophobic cavity structure of β-cyclodextrin enable guest encapsulation. Therefore, miRNAs gained hydrophobicity via conjugation with cholesterol or cationic bovine serum albumin (cBSA) enable to bind β-CD cavities to form CD-AD hyaluronic acid (HA). This on-covalent binding also enhances cellular uptake efficiency and miRNA release kinetics (Figure 8B) [144,160,166]. Other hydrogels, such as Pluronic F127, are also suitable for this mechanism [160]. It is worth noting that the self-assembly dynamics and release rates are limited by the ratio and affinity of host–guest molecules, making precise regulation difficult, which requires further in-depth research.

- Peptide self-assembling hydrogels

The self-assembled peptides (SAPs) allowing spontaneous formation of the three-dimensional network without chemical or physical treatment makes them versatile tools for minimally invasive surgery and rapid tissue repair. Among the most widely studied self-assembled hydrogel, peptide Ac-(RADA)4-NH2 (RADARADARADARADA-NH2) is a repeating sequence of arginine (R), alanine (A), and aspartic acid (D) arranged as alanine-arginine-aspartate (RAD) units [167,168]. This sequence design facilitates peptide interaction, enabling hydrogel formation with nucleic acids distributed inside (Figure 8C) [167]. In an aqueous environment, RAD peptides favor β-sheet structures due to hydrogen bonding between aspartic acid side chains. The β-sheets structure is critical for self-assembly and hydrogel network formation. Additionally, arginine residues carry positive charges, while aspartic acid residues are negatively charged, further stabilizing the β-sheet structure through electrostatic interactions and promote peptide chains aggregation. Meanwhile, alanine residue provide hydrophobicity, enhancing hydrophobic interaction between peptide chain to reinforce the self-assembled structure [195,196,197]. When exposed to ions, RAD peptide hydrogels undergo ion-induced cross-linking, enabling rapid in vivo gelation while maintaining biocompatibility and degradability (Figure 8D) [168]. In nucleic acid drug delivery applications, SAPs bind nucleic acid drugs via hydrogen bonding and electrostatic interactions, achieving high drug loading capacity (Figure 8D). By non-covalently conjugating the stem cell homing peptide SKPPGTSS (Figure 8C), SAPs can also recruit synovium-derived MSCs (SMSCs) to osteoarthritis injury sites, promoting cartilage regeneration [167,168]. In situ, self-assembled peptide hydrogels mimic the ECM properties, providing a conducive environment for cell attachment, proliferation, and differentiation [168]. This supports localized drug delivery and sustained release. The functionality of these hydrogels depends on precise amino acid sequence design to prevent enzymatic degradation and physical washing in physiological condition.

### 5.2. Three-Dimensional Delivery Systems for Nucleic Acid Therapy

Three-dimensional tissue engineering scaffolds provide an ideal platform for nucleic acid delivery while offering spatial support for cellular growth and differentiation. Their porous architecture enables cell infiltration and adhesion, while surface chemistry and topography critically regulate cellular behavior. This integrated functionality enhances nucleic acid therapies for tissue repair and regeneration. Strategic 3D design enables precise drug targeting through scaffold geometry tailored to anatomical features of specific tissues. Such innovations in scaffold design and their clinical applications are highlighted in Table 2.

#### 5.2.1. Scaffold Structure and the Preparation Method

Based on material composition, morphology and the preparation method, tissue engineering scaffolds can be categorized into three types: non-printed scaffolds for padding, 3D-printed porous braided scaffolds, and 3D scaffolds with special structures.

- Non-printed scaffolds for padding

Scaffolds for padding are designed to address the challenge of irregular tissue damages, particularly bone tissue. Injectable hydrogels, while capable of conforming to irregular cavities, often lack of sufficient mechanical strength. Non-3D-printed scaffolds for padding overcome this limitation by directly filling the defect site. Theses scaffold are typically fabricated from materials like collagen, with lyophilization [209,218]. For instance, S.Elangovan et al. utilized collagen scaffolds to deliver PEI–pDNA complexes to BMSCs, effectively promoting bone tissue formation [206]. However, the inherent mechanical properties of collagen may be insufficient for supporting the repair of large or load-bearing bones. Moreover, the degradation rate of collagen might not align with the pace of bone regeneration, potentially compromising the structural support for newly formed bone. To address these issues, collagen is often chemically cross-linked with other materials, like hydroxyapatite (HA), using lyophilization technology. HA, a highly rigid mineral, possesses the ability to promote osteoblast proliferation and differentiation. When combined with collagen, it significantly improves the mechanical strength and stability of the scaffolds. Additionally, chemical cross-linking improves the scaffold’s pore structure, increases the interconnectivity, and promotes cell adhesion, growth, and differentiation [72,205,207,219]. In summary, through the use of lyophilization technology and chemically cross-linking, non-3D-printed fillable scaffolds can be endowed with good mechanical properties, suitable degradation rate, and osteogenic capability.

- 3D-printed porous braided scaffolds

**Figure 9:**
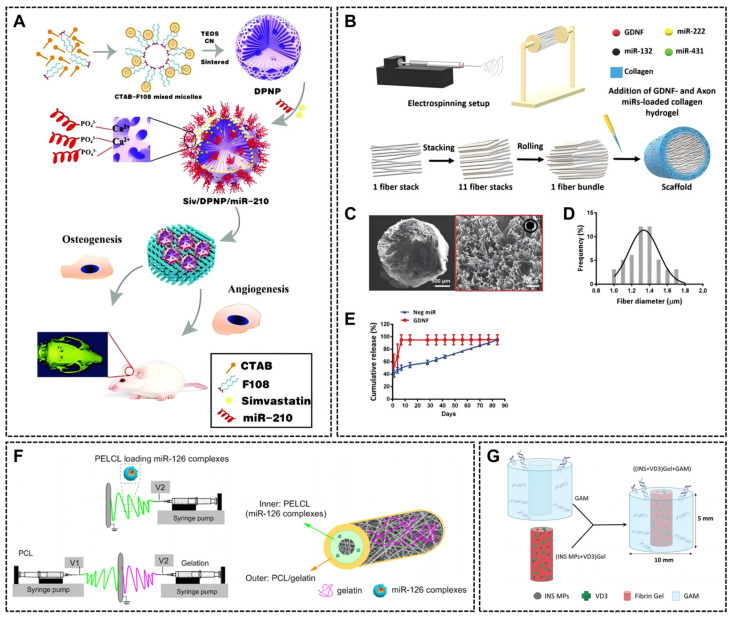
Advances in 3D printing: porous braided scaffolds and bionic structured scaffolds. (**A**) Schematics illustrating the preparation of a 3D-printed porous braided scaffold loaded with Siv/DPNP/miR-210 and its application for bone regeneration. Reprinted with permission from [129]. Copyright 2021, Royal society of chemistry. (**B**) Scaffold fabrication schematic diagram. (**C**,**D**) SEM imagery illustrating the complete scaffold alongside a magnified view of the PCL electrospun fibers (highlighted in red box). A total of 50 fibers were analyzed, yielding an average diameter of 1.35 ± 0.19 µm. The black arrow, positioned in the top right corner of (**C**), denotes the orientation of the fibers. (**E**) Cumulative release of Neg miRNA and GDNF over time [198]. Copyright 2021, Wiley-VCH. Licensed under CC BY 4.0. (**F**) Schematic representation for preparation of the bilayer electrospun membranes used in vascular tissue engineering [199]. Copyright 2016, Elsevier. (**G**) Schematic illustration of the composite scaffolds ((INS MPs+VD3) Gel+GAM) [99]. Copyright 2020, Wiley-VCH.

3D-printed porous braided scaffolds provide a unique platform for nucleic acids. J. Liu et al. used Direct Ink Writing (DIW) to fabricate β-tricalcium phosphate (b-TCP) scaffolds with tailored pore structures. These scaffolds were subsequently immersed in a solution containing drug carrying nanoparticles (DPNPs), enabling nucleic acid drugs loading (Figure 9A) [129]. Another approach involves encapsulating nucleic acids within hydrogels prior to scaffold loading. H. Hu et al. employed Digital Light Processing (DLP) printing technology and a sintering process to fabricate bioglass scaffolds. They also prepared GelMA/nanoclay hydrogels, soaked the scaffolds in a solution containing miRNA-sEVs, and then applied 365 nm ultraviolet radiation for 2 min to form a coating of sEVs films [216]. Compared with conventional fabrication techniques, 3D printing enables the creation of more complex and precise scaffold architectures. However, the coating method used for nucleic acids loading necessitates prior scaffold preparation. This type of scaffold is relatively uncommon as small molecule nucleic acids and their carriers are loaded physically, which may not ensure sustained and sequential release.

- 3D scaffolds with bionic structures

Bionic 3D scaffold design depends on the mechanical properties of target tissues. Morpho-functionally, these scaffolds are classified as 3D fiber–hydrogel or collagen composites or hydrogel composites.

Fiber–hydrogel/collagen composites effectively repair spinal cord and vascular tissues. The spinal cord’s white and gray matter contain orderly nerve bundles within a complex ECM providing mechanical support and cellular regulation. Spinal cord injury (SCI) repair thus requires replicating nerve bundle alignment and ECM properties. N. Zhang et al. utilized aligned polycaprolactone (PCL) fibers to mimic white matter axon arrangement, directing regenerating axon growth. These fibers were embedded in a collagen hydrogel matrix loaded with glial cell line-derived neurotrophic factor (GDNF) and miRNAs (miR-132/222/431). This structure promoted axon regeneration and neuronal functional recovery (Figure 9B–D), with sustained release of the GDNF over 1 week and miRNAs over 3 months, respectively (Figure 9E) [198]. Polymers like PCL, PCL-DPP-PCL, and PCLEEP are often used for the internal fibers, while hydrogels form the outer layer [200,201]. Electrospinning allows the fabrication of nanofibers with specific arrangements and diameters, facilitating cell attachment, migration, and differentiation. This structure is also suitable for vascular tissue engineering. For instance, F. Zhou et al. used PELCL as the inner layer, prepared via emulsion electrospinning and PCL mixed with gelatin were used as the outer layer, prepared by dual-power electrospinning (Figure 9F). The PELCL inner layer delivered miRNA-126 to regulated the response of vascular endothelial cells (VECs), while the outer layer provided mechanical stability and supported cell attachment and growth [199].

Hydrogel composite structure shares similarities to 3D fiber–hydrogel/collagen composites (Figure 9G). B. Khorsand et al. used a fibrin gel as an inner layer to deliver insulin (INS) and active vitamin D3 (VD3) metabolites to improve bone healing in diabetes models without affecting systemic glycemic parameters. The outer layer consisted of a collagen matrix combined with a PEI-(pBMP-2+pFGF-2) nanocomposite. The pDNA encoding BMP-2 and FGF-2 from non-viral gene delivery worked synergistically to promote bone regeneration. The multi-layered structure and multi-drug delivery scaffold enhanced bone formation and healing in diabetes models [99].

While 3D scaffolds with bionic structure offer superior biomimetic properties, their preparation is often complex process. The effectiveness of composites materials requires further investigation. Additionally, producing sheet polymeric fiber scaffolds may involve more complex processes and higher costs compared to conventional drug delivery systems. Although drug release can be controlled by adjusting the coating, precise control in complex biologically environments remain a challenge. The clearance mechanisms of drugs and materials post-release also need to be carefully considered to avoid long-term side effects.

#### 5.2.2. Design for Nucleic Acid Release

The 3D structure of tissue engineered scaffolds is a key factor in designing nucleic acid release systems. As mentioned previously, injectable hydrogel-based designs are common approach. 3D scaffolds often consist of multiple materials beyond hydrogels. These scaffolds typically have porous structures that facilitate the loading and release of nucleic acids, allowing them to penetrate the scaffold to interact with cells. 3D architecture makes surface modification of scaffolds easier. In addition to traditional physical encapsulation, nucleic acids and their vectors can be adsorbed on scaffolds in the form of coatings.

The nucleic acid release can be designed to align with the tissue regeneration processes. For instance, a collagen sponge can be mineralized in simulated body fluid (SBF) to form a calcium-deficient hydroxyapatite layer. A complex of pDNA encoding bone morphogenetic protein-2 (BMP-2) and polyethylenimine (PEI) is embedded in the CDHA coating. The PEI-pBMP-2 complex to co-precipitate with the minerals in the SBF to form an inner layer. Subsequently, the pDNA encoding fibroblast growth factor-2 (FGF-2) also complexed with PEI is embedded in the CDHA coating to form an outer layer. This double-layer structure design allows for sequential release of the two growth factors. BMP-2 is responsible for promoting osteogenic differentiation of bone marrow MSCs (BMSCs), while FGF-2 is responsible for enhancing cell proliferation and angiogenesis. The sequential release mimic the natural growth factor release pattern during bone healing [203].

In addition to sequential release, 3D scaffolds can also be designed as stimulus-responsive systems, although it is less common. For instance, PHTB (TA-siRNA) hydrogels were synthesized from polyvinyl alcohol (PVA), human-like collagen (HLC), tannic acid (TA), and borax through reactive liquid molding. Borate and hydrogen bonds are crucial for dynamic cross-linking. TA-siRNA nanogels, formed by a self-assembling of TA and siRNA, are loaded into the hydrogel via hydrogen bonds and borax-mediated borate bonds. In diabetic chronic wounds, high oxidative stress due to abnormal glucose and lipid metabolism lead to elevated level of reactive oxygen species (ROS) secreted by macrophages and neutrophils. Borate bonds in PHTB (TA-siRNA) hydrogels are oxidized by hydrogen peroxide at normal physiological pH and temperature. As ROS level rises, borate bonds break, reducing cross-linking density, disintegrating the network structure, increasing pore size, and expanding pore distribution. This change facilitates the release of TA-siRNA nanogels [213].

In summary, 3D nucleic acid drug scaffolds mainly achieve sustained release through self-degradation, pore size, and porosity. However, research on its stimulus-responsive release remains limited. Scaffold topology, particularly pore size and interconnectivity within 3D matrices, critically determines both cellular infiltration and nucleic acid drug release which are critical for therapeutic efficacy. Larger pore diameters (>100 μm) generally facilitate enhanced stem cell recruitment by promoting cell migration, nutrient diffusion, and vascular ingrowth [220]. Conversely, smaller pores (<50 μm) offer higher surface area to volume ratios, increasing nucleic acid loading capacity but potentially restricting cell penetration. This architecture also governs release kinetics: smaller pores create more tortuous diffusion pathways, prolonging sustained release of nucleic acids (e.g., pDNA and siRNA) and protecting them from premature degradation [221]. However, excessive confinement can hinder cell–scaffold interactions essential for transfection. Optimal pore design must therefore balance these competing demands, ensuring sufficient cell ingress while modulating release profiles to match therapeutic windows. Furthermore, pore geometry influences paracrine signaling gradients, indirectly modulating stem cell homing and activation [222]. While 3D structures are effective for localized delivery, implantation induced trauma and complete degradability are concerns requiring further investigation. Future scaffold optimization should integrate computational modeling of pore networks with empirical studies to precisely tailor topology for specific regenerative applications.

### 5.3. Sheet-Like Delivery Systems for Nucleic Acid Therapy

The sheet-like delivery systems offer another approach for nucleic acid therapy, typically taking the form of dressings or patches. They are particularly suited for treating superficial tissue damages and shallow wound.

#### 5.3.1. Scaffold Structure and the Preparation Method

The sheet-like scaffolds are primarily composed of hydrogels or polymer fibers. They can be categorized into single-layer and multi-layer types. Additionally, there are distinct layer-by-layer (LbL) thin sheets. An overview of the designs and applications of sheet-like delivery systems is shown in Table 3.

- Hydrogel 3D sheet-like scaffolds

**Figure 10:**
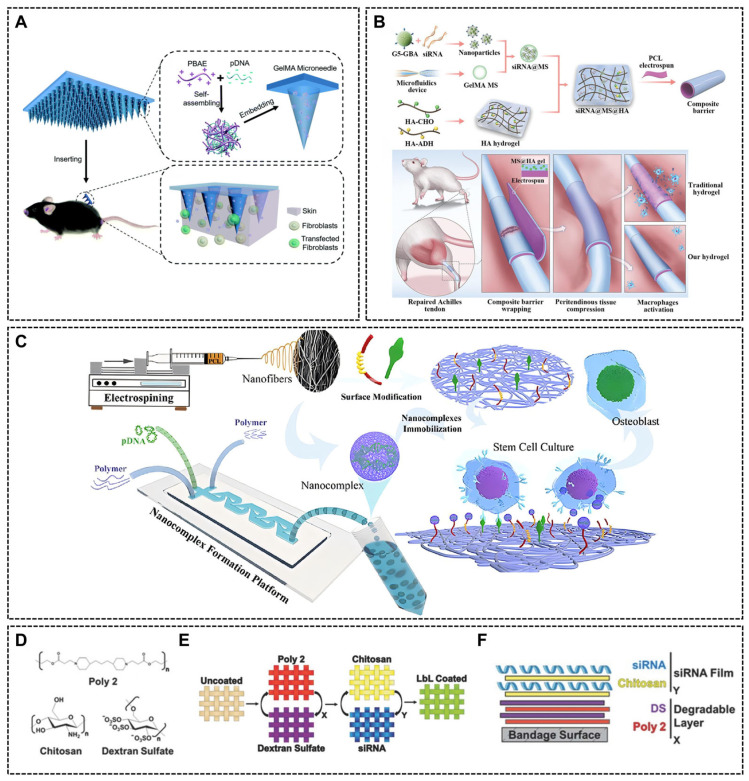
Structure and preparation method of sheet-like scaffolds. (**A**) The components and application of MN/PBAE/DNA [107]. Copyright 2020, Royal society of chemistry. (**B**) Fabrication procedure of the siRNA@MS@HA hydrogel–electrospun membrane, and the implantation into rat injured tendon site [223]. Copyright 2022, Wiley-VCH. (**C**) A schematic representation of a Tesla micromixer to make chitosan-based gene delivery nanocomplex and the electrospinning processes used to make poly(ε-caprolactone) (PCL) nanofibers modified by nanocomplex [86]. Copyright 2020, Elsevier. (**D**) Chemical structures of Poly2, chitosan and dextran sulfate. (**E**) The hierarchical structure of LbL films into a single coating. The first (X) film is a hydrolytically degradable undercoating, while the second (Y) film contains the siRNA to be delivered. (**F**) Architecture of hierarchical LbL [235]. Copyright 2016, Wiley-VCH.

Monolayer hydrogel sheets serve as critical platforms for skin tissue repair, providing physical infection barriers when applied directly to wounds. For instance, S.C. Tao et al. developed chitosan hydrogel dressings loaded with miR-126-3p-enriched synovial MSC exosomes (SMSC-exos). These enabled sustained exosome release and accelerated healing of full-thickness diabetic wounds in rats. Beyond establishing moist microenvironments, the chitosan matrix contributed essential hemostatic and antimicrobial functions, particularly vital for chronic diabetic wound healing [52]. To address deeper tissue delivery, microneedle patches have been explored for transdermal administration. These patches are able to penetrate the stratum corneum, facilitating deeper drug delivery with minimal pain [232,233]. M. Qu et al. reported a transdermal microneedle patch for delivering PBAE/DNA nanoparticles through a cross-linkable GelMA matrix. The GelMA microneedle patches can penetrate the epidermis to reach the targeted cells (Figure 10A) [107]. The performance of sheet hydrogels can be enhanced by combining with other polymers. For instance, C. Cai et al. created a composite film by integrating hydrogels with PCL (polycaprolactone) electrospun nanofibers to form that enhanced adhesion to peritendinal tissues (Figure 10B) [223]. In another study, scaffolds combing a collagen-chitosan layer with a silicon membrane can control water loss and prevent bacterial entry, winning time for transplantation of ultra-thin skin grafts [225].

In summary, hydrogel sheets are of great significance for skin tissue engineering. However, further research is needed for their effectiveness, particularly in reducing scarring while promoting skin regeneration.

- Polymer fiber 3D sheet-like scaffolds

Polymer fiber 3D sheet-like scaffolds are primarily created by electrospinning, which involves melting a polymer to solution and ejecting it with a high voltage to form nonwoven fiber pads. This technology is widely used in tissue engineering and biomedicine [234]. A. Malek-Khatabi et al. fabricated a polycaprolactone (PCL) nanofiber scaffold for the delivery of chitosan–pDNA complexes by electrospinning. The pDNA and chitosan were mixed and modified by a microfluidic mixer to form nanocomplexes. This nanofiber structure provides a large surface area for pDNA loading and supports cell growth and penetration in vivo and in vitro (Figure 10C) [86]. The surface of these scaffolds can also be easily modified by polymers and hydrogels. For instance, Y. Wang et al. prepared PLLA (poly-L-lactic acid) and silk fibroin parallel fiber (PSPF) films via electrospinning and modified their surface with PGA. The PGA coating gave PSPF@PGA fiber membrane a negative charge, facilitating its binding to RCP/pDNA nanoparticles through electrostatic attraction. PGA coating may also improve the membrane’s compatibility with cells, promoting cell adhesion and proliferation. This patch-style scaffold serves as a local gene delivery platform for delivering the pJUN plasmid to Schwann cells in damaged nerve regions [228].

- Layer-by-layer thin sheets

Layer-by-layer (LbL) thin sheets are another type of sheet-like scaffolds. This technique involves constructing multi-layer thin sheet structures by alternating layers of oppositely charged materials, allowing for precise control of the thickness and composition. Mandapalli, P. K et al. created an LbL film by alternately adsorbing chitosan and sodium alginate solutions on a glass template, forming a 15-layer bilayer structure loaded with EGF and TGF-β siRNA. The film exhibited excellent mechanical properties, supported the adhesion and growth of A431 epidermal keratinocytes, and inhibited bacterial growth in vitro. In a C57BL/6 mouse incision wound model, the film accelerated wound healing, reduced collagen deposition and significantly promoted re-epithelialization by releasing constantly EGF and TGF-β [231]. In another study, siRNAs were encapsulated on dressings using LbL technology. The scaffolds consisted of a degradable layer poly(β-aminoester)2 (Poly2), then dextran sulfate (DS) in the middle, and an upper siRNA-containing chitosan layer. Poly2 is a well-established hydrolytically degradable polycation that facilitates water-based erosion in multi-layer films. The anionic DS and the cationic chitosan form stable multi-layer membrane structures through electrostatic interactions (Figure 10D–F). Poly2, containing β-amino ester groups with strong cationic properties, forms complexes with negatively charged chondroitin sulfate. Its molecular chain includes ester bonds (-COO-) and amino ester bonds (-NH-CO-O-), which hydrolyze in physiological environments for the slow siRNAs release. This structure effectively protects siRNA and enables precise membrane construction. LbL technology protects siRNAs during delivery, reducing digestion risks [235]. Recently, LbL strategies have also been extended to effective intracellular mRNA delivery, a topic of growing interest. For example, positively charged poly-L-lysine (PLL) and poly-L-ornithine (PLO) form mRNA polyplexes that are arranged in an LbL fashion with negatively charged layers. These assemblies are stable at extracellular pH (remaining net negatively charged), but they acquire a net positive charge in the acidic endosomal environment. This pH-triggered charge reversal facilitates endosomal membrane disruption and promotes cytosolic release of mRNA [236]. LbL thin sheets are advanced scaffolds that require further research to fully explore their potential.

#### 5.3.2. Design for Nucleic Acid Release from Sheet-Like Scaffolds

The design of nucleic acid release for sheet-like scaffolds is another important aspect to consider. The swelling and degradation of hydrogels have been discussed earlier in Section 5.1.1, and similar principles apply to sheet-like hydrogels as injectable hydrogels. Sheet hydrogels can sometimes be designed to be stimulus responsive [86,223]. For instance, A. Malek-Khatabi utilized chitosan as a cationic carrier to form nanocomposites (NCs) with plasmid DNA (pDNA) through electrostatic interactions. These NCs were then linked to the MMP-sensitive peptide sequence (GCRDGPQG↓IAGQDRCGC) on the surface of PCL nanofibers using cross-linker Sulfo-LC-SPDP. This design allows for the substantial release of NCs in an environment containing MMP-2 enzyme [86]. The release mechanisms for both hydrogels and polymeric fiber sheet-like scaffolds are primarily based on degradation. In particular, multi-layer sheet-like scaffolds can achieve the continuous and sequential release of nucleic acid drugs through LbL degradation [237]. S. A. Castleberry et al. designed a degradable vector system consisting of a poly(β-aminoester)2 and dextran sulfate (DS) bottom layer, and an upper siRNA-containing chitosan (Chi) layer. In this system, the release of siRNAs is controlled by adjusting the number of layers of the LbL membrane, and the degradation rate of the underlying layer determines the release rate and duration of siRNA [235]. In general, there are relatively few designs for sustained nucleic acid release from sheet-like scaffolds. Future research should focus on developing more advanced sheet-like 3D scaffolds to achieve better control over the nucleic acid release characteristics.

## 6. Clinical Translation Prospect and Challenges of Nucleic Acid Delivery Systems

Injectable scaffolds, 3D structural scaffolds, and sheet-like scaffolds represent distinct nucleic acid delivery platforms with significant potential for clinical translation in regenerative medicine. Their unique structural designs enable targeted repair strategies across diverse tissue types.

- Injectable Hydrogels

Offering minimally invasive catheter-assisted delivery [238], injectable hydrogels are particularly suited for deep tissue repair (e.g., myocardial infarction). They provide mechanical support to mitigate extracellular matrix defects and ventricular dilation. Tunable mechanical properties contribute to functional improvements, including enhanced left ventricular ejection fraction (LVEF; increases ~10% reported [126]) and angiogenesis. Furthermore, they serve as versatile platforms for sustained nucleic acid release, antioxidant integration, and stimuli-responsive design.

- 3D Structural Scaffolds:

Characterized by high biomimicry and load capacity, 3D scaffolds demonstrate significant potential for whole organ and bone regeneration. Their porous architecture facilitates cell migration, vascularization, and integration with mineralized tissues [206]. The inherent customizability supports translational progression into clinical applications, such as addressing long bone nonunion (e.g., ClinicalTrials.gov ID: NCT02323451). Future advancements are anticipated through integrating 3D printing with intelligent release systems for personalized fracture repair platforms.

- Sheet-Like Scaffolds:

Engineered for superficial large wound repair (e.g., burns and diabetic foot ulcers), these scaffolds leverage biomimetic structures coupled with molecular regulation. Key attributes include stretchability, skin adhesiveness, and anti-infective properties, paving the way for patient-specific precision therapies in wound healing.

Despite this promise, translating any delivery system necessitates overcoming critical challenges: (1) GMP manufacturing challenges in scaffold sterilization, nucleic acid stability, and batch consistency; (2) scalability limitations of productions; (3) administration pathways; and (4) commercial viability.

### 6.1. GMP Manufacturing Challenges

Good Manufacturing Practice (GMP) compliance is paramount. Key hurdles for GMP application include (1) Sterilization: Traditional methods (e.g., autoclaving) risk damaging heat-sensitive polymers. Alternatives like gamma irradiation or ethylene oxide require optimization to ensure sterility without compromising scaffold integrity. (2) Nucleic Acid Stability: Susceptibility to nucleases, pH shifts, and temperature fluctuations demands protective strategies during manufacturing, storage, and delivery. These include nuclease-free environments, stabilizing formulations (e.g., nanoparticle encapsulation), and stringent environmental controls. (3) Batch Consistency: Variations can arise in scaffold properties (pore size, mechanical strength), nucleic acid loading efficiency, and release kinetics. Ensuring consistency necessitates rigorous process control, including standardized raw materials, calibrated equipment, and comprehensive in-process testing.

### 6.2. Scalability Limitations

Scaling production presents significant obstacles: (1) Process Complexity: Personalized nucleic acid preparation, encapsulation, scaffold fabrication, and 3D bioprinting involve intricate steps challenging to automate for large-scale output. (2) Bioprinting Constraints: Current bioprinting speeds are often inadequate for large scaffolds. High costs of specialized bio-ink and support materials further limit scalability. (3) Consistency at Scale: Maintaining uniform mechanical properties and cell compatibility across numerous scaffolds during mass production remains difficult.

### 6.3. Regulatory Pathways (NMPA/FDA/EMA Frameworks)

Combination products (biomaterials + nucleic acids) face complex regulatory landscapes. Agencies (NMPA, FDA, EMA) classify products based on the primary mode of action: (1) Nucleic acid-driven therapeutics may be regulated as biologics (e.g., viral vectors), demanding extensive safety and efficacy data. (2) Requirements universally include comprehensive data on scaffold biocompatibility, nucleic acid stability (in storage and in vivo), and immunogenicity risk assessment. (3) The approval pathway involves pre-application consultations with regulatory bodies, followed by a detailed registration application encompassing product development, manufacturing, biocompatibility, stability, immunogenicity, preclinical studies, and clinical trial results.

### 6.4. Commercial Viability Metrics

Cost-per-dose analysis is central to commercial feasibility: (1) Viral Vectors: (e.g., adenovirus, AAV, and lentivirus) offer high transfection efficiency but incur substantial costs due to complex cell culture, purification, and stringent quality control. Safety concerns (insertional mutagenesis, immunogenicity) may necessitate additional costly safety testing. (2) Non-Viral Vectors: (e.g., lipid/polymer-based systems) generally exhibit lower immunogenicity and potentially lower production costs via simpler chemical synthesis. However, lower transfection efficiency often requires higher doses, potentially offsetting cost advantages. Viability assessments must include raw materials, manufacturing, storage, and distribution costs. Enhancing non-viral vector efficiency and developing cost-effective manufacturing are crucial for improving commercial prospects.

## 7. Conclusions and Future Perspectives

The application of nucleic acids in regenerative medicine treatments has expanded rapidly over the past decade. While small RNAs such as miRNA, siRNA, and pDNA remain central to therapeutic strategies, new emerging lncRNAs have been gaining the attention of scientists for their regulatory roles in tissue repair. Despite this progress, researchers focus predominantly on RNA interference mechanisms in the field of regenerative medicine, while other nucleic acids modalities, particularly those involving epigenetic regulation, are underexplored and need to be studied further. In recent years, CRISPR-Cas9, a breakthrough gene-editing technology, has shown promise in addressing genetic disorders, such as hereditary sarcopenia, and has begun to take off [239]. But this technique requires further study on broader regenerative applications. There is still a critical gap in understanding tissue specificity and metabolic heterogeneity within injured microenvironments and target tissues, where dynamic molecular interactions necessitate multi-omics approaches to decode patient-specific expression profiles. Moreover, nucleic acids can also play an indirect role. Research has shown that the scaffold is prepared by employing a 3D tetrahedral framework nucleic acid (tFNA) covalently cross-linked to methacrylated hyaluronic acid (HAMA) via thiol–maleimide chemistry, forming a photocurable composite hydrogel (HA-p3T) for in situ capture and enrichment of endogenous BMP-2. By this design, tFNA serves as a fixed functional module. No nucleic acid release occurs, but it realizes stable BMP-2 binding and sustained release [240]. This mechanism needs to be further researched.

For the intracellular delivery of nucleic acid drugs, developing polymeric mRNA delivery systems must consider the critical observation that mRNA is highly susceptible to degradation, even when encapsulated within stable complexes [241]. Therefore, future designs should focus on enhancing not only complex stability but also protective mechanisms that safeguard mRNA integrity throughout delivery. Such advances are crucial for enhancing the efficacy and therapeutic potential of mRNA-based therapies. Specifically, designs must overcome four key barriers: the degradation of nucleases, charge repulsion, endosomal entrapment and off-target effects. Viral vectors, such as adenovirus, adeno-associated virus, and lentivirus, remain the most frequently used technology for high transfection efficiency. However, safety concerns have led us to focus on non-viral nanocarriers, including organic and inorganic materials. Exosomes naturally derived from stem cells, exhibit unique biocompatibility but face challenges due to their complex cargo composition, which obscures mechanistic insights into synergistic nucleic acid delivery [242,243]. The mechanism needs to be further clarified. Synthetic alternative organic materials, including lipids, peptides, and polysaccharide derivatives, should be noticed for their extraordinary biocompatible. In addition, inorganic materials, like metals and nanoceramic, offer modular design flexibility to enhance payload protection and cellular uptake of nucleic acid drugs. However, their long-term biocompatibility, biodegradability, and potential bioaccumulation require rigorous evaluation.

Beyond intracellular hurdles, tissue-targeted delivery also needs to be considered. Blood circulation and physiological clearance mechanisms often eliminate nucleic acids from targeted site, prompting the development of scaffold-based localization strategies [244]. Injectable hydrogels enable minimally invasive delivery but suffer from limited mechanical stability [245]. Conversely, 3D-printed scaffolds provide structural precision yet face challenges in nucleic acid drugs leading efficiency, complex preparation processes and weak scalability [246]. In vivo 3D printing, an emerging technique, bypasses the shortcomings of in vitro 3D fabrication and has demonstrated feasibility. Recent research has successfully delivered nucleic acids through in vivo 3D printing to repair craniofacial defects [83]. Nevertheless, biological interactions between printed scaffolds and dynamic microenvironments remain poorly understood. This review systematically examines biomaterial-based nucleic acid delivery systems for regenerative medicine applications. We analyze three interconnected strategic platforms: (1) viral and non-viral vectors, (2) engineered nanocarriers (organic and inorganic), and (3) 3D scaffold-based systems. A comprehensive comparative analysis of these platforms is presented in Table 4: Scaffold Platform Comparative Matrix, which synthesizes their relative advantages, limitations, and optimal therapeutic applications. This matrix specifically evaluates: (1) viral versus non-viral vector systems; (2) organic versus inorganic nanocarrier designs; (3) scaffold-mediated versus systemic delivery approaches. Nanocarrier design focuses on physicochemical modifications, such as surface charge modulation and ligand conjugation, in order to overcome biological barriers and enhance intracellular transport, while scaffold fabrication methodologies emphasize spatiotemporal control of nucleic acid release through stimuli-responsive materials or biomechanically tailored architectures.

Despite these innovations, knowledge gaps in this area remain, including how to achieve precise spatiotemporal control of payloads like lncRNAs within complex, dynamic microenvironments, and how to prevent rapid clearance and immune activation while maintaining transfection efficiency. Emerging modalities such as CRISPR-activated lncRNAs (e.g., DANCR for bone regeneration) highlight the potential of lncRNAs as epigenetic regulators, yet their in vivo trafficking, cell-type specificity, and downstream network effects remain poorly mapped [247]. In parallel, in situ 3D bioprinting—where biomaterial “bio-inks” laden with nucleic acids or cells are printed directly into lesions—offers patient-personalized geometry, but faces bottlenecks in vascularization of thick constructs, resolution limits for nanoscale gene delivery, and lack of standardized, immune-evasive bio-inks [248]. Future research should therefore integrate “smart” stimuli-responsive biomaterials that release lncRNA cargos on demand with real-time imaging feedback, develop hybrid nanocarriers that co-deliver lncRNAs and pro-angiogenic factors during printing, and establish high-throughput in situ screening platforms to delineate lncRNA dose–function relationships across tissue types. Addressing these challenges will convert lncRNA-based epigenetic engineering and in situ 3D printing from proof of concept into next-generation regenerative therapies.

Translational challenges persist, including long-term biocompatibility of synthetic vectors, scalability of scaffold manufacturing, and dynamic adaptation to heterogeneous tissue microenvironments. By highlighting advances in exosome biohybrids, scaffold biofunctionalization, such as CRISPR-Cas9-loaded matrices, and in situ 3D bioprinting technique, this work proposes a multidisciplinary roadmap to bridge preclinical efficacy with clinical safety, accelerating the translation of nucleic acid therapies into mainstream regenerative practice.

## Figures and Tables

**Table 1:** Designs and applications of injectable delivery systems.

Design	DNA/RNA	Target Sites/ Additive Fragments	Vectors	Scaffold Materials	Application	Reference
Light-responsive release	miR-26a-5p	N/A	Cholesterol	PEG	Calvarial bone defect	[148]
PH-responsive release	miR-21-5p	SPRY1	MSNs	α-CD, PEG-CHO	Myocardial infarction	[126]
	siRNA	STING	PAMAM	HA-CHO	Intervertebral disc degeneration	[114]
	siRNA	P65	G5 PAMAM-PBA	OG, ADH-GCA	Intervertebral disc degeneration	[149]
Enzyme-responsive release	miR-29a	Col I/Col III/TGF-β	polyplex micelles	Peptide CGPLGVRGC, eight-arm PEG Maleimide (hexaglycerol)	Intervertebral disc fibrosis	[150]
	miR-99a-3p	ADAMTS4	ADSCs exosome	PEG-HA	Osteoarthritis	[151]
	siRNA	MMP2	Cholesterol	HA	Myocardial infarction	[145]
Photosensitive hydrogel	miR-17-5p	PTEN/p21	hUC-MSCs sEVs	GelMA	Diabetic wound healing	[152]
	miR-26a	Tob	rats BMSCs exosome	GelMA-CSMA	Calvarial bone defect	[153]
	miR-146a	TRAF6/NF-κB	MSNs	GelMA	Mandibular defect	[128]
	miR-146a	N/A	CNPs	SBMA, HEMA, PEGDMA	Diabetic wound healing	[134]
	miRNA-223	N/A	HA	GelMA	Wound healing	[154]
	siRNA	VEGFa	LNPs	GelMA	Cartilage regeneration	[60]
	siRNA	PTEN/MIF	LNPs	GelMA	Spinal cord injury	[155]
	siRNA	noggin	SA/Chol sterosome	MeGC	Calvarial bone defect	[156]
	siRNA	Wwp1	pDMAEMA-b-p(DMAEMA-co-PAA-co-BMA)	PEG-PLA-DM	Bone fracture	[157]
Temperature-sensitive hydrogel	miRNA-24-3p	N/A	rabbit ADSCs exo	DEGMA-HA	Corneal epithelium	[158]
	miR-26a	N/A	RALA	Cs-g-PNIPAAm	Calvarial bone defect	[68]
	miR-29b	PDGFRβ	Lentiviral vectors	Matrigel	Skin wound healing	[159]
	miR-29b	N/A	BSA	PF-127	Renal interstitial fibrosis	[160]
	miR-222	NLK	MSNs	PEG–PLGA–PNIPAM tri-block polymer	Mandibular defect	[127]
	miR-223	PDGFRβ	Cholesterol	Pluronic gel	Arterial injury	[161]
	miR-615	LINGO-1	N/A	PF-127	Neuron damage	[162]
	siRNA	PERK	Lentiviral vectors	PLGA–PEG–PLGA tri-block polymer	Post-angioplasty reendothelialization	[163]
	siRNA	PPARc	bPEI	PLGA–PEG–PLGA tri-block polymer	Alcohol-induced osteonecrosis	[102]
	shRNA	LINGO-1	Lentiviral vectors	PF-127	Ventral root avulsion	[20]
	shRNA	LINGO-1	Lentiviral vectors	PF-127	Spinal cord injury	[164]
	shRNA	ACE	Lipofectamine 2000	Dex-PCLHEMA/PNIPAAm	Myocardial infarction	[165]
	pDNA	BMP-2	Chitosan nanoparticles	Chitosan	Calvarial bone defect	[84]
	pDNA	BMP-2	Chitosan nanoparticles	Chitosan	Endogenous repair of the periodontium	[81]
Host–guest self-assembling hydrogels	miR-302	Mst1/Lats2/Mob1	Cholesterol	HA	Myocardial infarction	[144]
	miR-302	Hippo	Cholesterol	Adamantane-HA, cyclodextrin-HA	Myocardial infarction	[166]
Peptide self-assembling hydrogels	miR-29b-5p	N/A	N/A	SAP hydrogel-conjugated peptide SKPPGTSS	Cartilage	[167]
	miR-145	N/A	N/A	Self-assembling RAD peptide hydrogel	Intimal hyperplasia post-vascular injuries	[168]
	pDNA	Klotho	Peptide NPs	KLDL hydrogel-conjugated peptide PFSSTKT	Osteoarthritis	[169]
Physical cross-linking	miR-22	MECP2	N/A	Laponite hydrogels	Vascular injuries	[170]
	miR-126/miR-146a	SPRED-1(miR-126), IRAK-1/TRAF6(miR-146a)	ADSCs exosome	Sodium alginate	Myocardial Infarction	[53]
	miR-146a	N/A	CNPs	CBMA or SBMA, HEMA	Diabetic wound healing	[137]
	miR-675	TGF-β1	hUC-MSCs exosome	Silk fibroin	Aging-induced vascular dysfunction	[171]
	siRNA	CAPG	Lipofectamine RNAiMAX	dECM	Cardiac fibroblast	[64]
	siRNA	MMP9	chitosan nanoparticles	Sodium alginate, 45S5 Bioglass^®^ powder	Diabetic wound healing	[172]
	siRNA	MMP9	N/A	HA, SA	Diabetic wound healing	[173]
	DNA	PDGF-B	adenovirus vectors	Collagen, polyvinyl alcohol sponge	Soft tissue repair	[38]
	pDNA	bFGF	PEI-PEG	alginate, HA	Vocal fold	[104]
Chemical cross-linking	miR-26a-5p	N/A	EC-Exosome	HA	Bone fracture	[54]
	miR-29a	HDAC4	mice BMSCs exosome	HB-PEGDA, SH-HA	Bone fracture	[174]
	miR-29b	N/A	N/A	SH-HA-PEGDA	Myocardial infarction	[175]
	miRNA-199a-3p	HOMER1/CLIC5	DSPE-PEG, TAT-peptide	ELP-HYD, HA-ALD	Myocardial infarction	[39]
	miR335-5p	DKK1	Tetrahedral DNA	Li-hep-gel	Steroid-associated osteonecrosis	[176]
	miR-5590	DDX5	SNAs	DNA hydrogel	Disc	[97]
	miRNA	COX	PLGA	HA	Tendon	[112]
	mRNA	VEGF-A/BMP-2	hAD-MSCs sEVs	PEGS-A	Femur critical-size defects	[55]
	siRNA	SPARC	N/A	Gelatin-tyramine (Gtn-Tyr) hydrogel	Anti-scarring therapy	[177]
	siRNA	Mstn	Invivofectamine^®^ 3.0	Silk fibroin	Skeletal muscle	[63]
	siRNA	DDIT4	G5-PAMAM	HA	Disc	[115]
	lncRNA HAR1B	KLF4	HUVECs exo	β-cyclodextrin derivatives, gelatin, chitosan	Diabetic wound healing	[24]
Physical and chemical cross-linking	DNA	BMP-7	Adenovirus vectors	Collagen, fibrinogen	Bone defects	[178]
Others	miR-335-5p	DKK1	LNPs	Silk fibroin	Calvarial bone defects	[179]
	miR-1825	NDUFA10	AAV vectors	Gelatin, silicate	Myocardial infarction	[180]
	siRNA	Rb1/Meis	Liposomes	Gelatin, silicate	Myocardial infarction	[65]

Annotation: PEG = polyethylene glycol, MSNs = mesoporous silica nanoparticles, α-CD = alpha-cyclodextrin, PAMAM = poly(amidoamine), HA = hyaluronic acid, PBA = phenylboronic acid, OG = oxidized dextran, ADH-GCA = adipic acid dihydrazide-grafted catechol-coupled gelatin, ADSCs = adipose-derived stem cells, hUC-MSCs = human umbilical cord-mesenchymal stem cells, GelMA = gelatin methacryloyl, BMSCs = bone marrow mesenchymal stem cells, CSMA = chitosan-methyl methacrylate, CNPs = cerium oxide nanoparticles, SBMA = [2-(methacryloloxy)ethyl]dimethyl-(3-sulfopropyl) ammonium hydroxide, HEMA = 2-hydroxyethyl methacrylate, PEGDMA = polyethylene glycol dimethacrylate, LNPs = lipid nanoparticles, SA = stearylamine, Chol = cholesterol, MeGC = methacrylated glycol chitosan, DMAEMA = dimethylaminoethyl methacrylate, PLA = poly(lactide), DM = dimethacrylate, DEGMA = di(ethylene glycol) monomethyl ether methacrylate, PNIPAAm = poly(N-isopropylacrylamide), BSA = cationic bovine serum albumin, PF-127 = pluronic F127, PEG-PLGA-PNIPAM = poly(ethylene glycol)-b-poly(lactic-co-glycolic acid)-b-poly(N-isopropylacrylamide), PLGA = poly(lactic-co-glycolic acid), PEI = polyethylenimine, bPEI = branched polyethylenimine, Dex-PCL-HEMA/PNIPAAm = dextran-poly(e-caprolactone)-2-hydroxylethylmethacrylate-poly(N-isopropylacrylamide), CBMA = 3-[[2-(methacryloyloxy)ethyl] dimethylammonio] propionate, dECM = decellularized extracellular matrix, EC-Exos = endothelial cell-derived exosomes, HB-PEGDA = hyperbranched polyethylene glycol diacrylate, SH-HA = sulfhydryl-modified hyaluronic acid, SH-HA-PEGDA = thiolated hyaluronic acid cross-linked with poly(ethylene glycol) diacrylate, DSPE = 1,2-distearoyl-sn-glycerol-3-phosphoethanolamine, TAT peptide = transactivator of transcription peptide, ELP = elastin-like protein, HYD = hydrazide, ALD = aldehyde, SNAs = spherical nucleic acids, hAD-MSCs = human adipose-derived mesenchymal stem cells, PEGS-A = PEGylated poly(glycerol sebacate) acrylate (PEGS-A), and PAMAM = polyamidoamine.

**Table 2:** Designs and applications of three-dimensional delivery systems.

Design	DNA/RNA	Target Sites/ Additive Fragments	Vectors	Scaffold materials	Application	Reference
3D scaffolds with bionic structure	miR-132/miR-222/miR-431	Rasa1 (miR-132)	TransiT-TKO	Collagen, PCL	Spinal cord injury	[198]
		PTEN (miR-222)				
		Kremen1 (miR-431)				
	miRNA-126	SPRED-1	N/A	PELCL, PCL, gelatin	Angiogenesis	[199]
	miR-219/miR-338	N/A	TransiT-TKO	PCLEEP, collagen	Central Nervous System Remyelination	[200]
	miR-219/miR-338	TNF-α, GFAP	TransiT-TKO	PCLEEP, collagen	Central Nervous System Remyelination	[201]
	pDNA	BMP-2, FGF-2	PEI	Collagen matrix, fibrin gel	Bone Formation in a Diabetic Model	[99]
	pDNA	TGF-β, NMNAT2	HA-GMA/PEI	PCL/HA-GMA	Sciatic nerve regeneration	[202]
	pDNA	BMP-2, FGF-2	PEI	Collaplugs	Calvarial bone defect	[203]
Non-3D-printed scaffolds for filling	pDNA	VEGF, PDGF	PEI	Collagen	Calvarial bone defect	[204]
	pDNA	BMP-2, VEGF	star-PLLs	Collagen, hydroxyapatite	Calvarial bone defect	[205]
	pDNA	BMP-2, VEGF	GET peptide	Collagen, hydroxyapatite	Calvarial bone defect	[72]
	pDNA	PDGF-B, VEGF	PEI	Collagen	Calvarial bone defect	[206]
	pDNA	BMP-2, VEGF	Chitosan	CHA	Calvarial bone defect	[207]
	pDNA	caALK6, Runx2	PEG-b-P[Asp-(DET)]	Calcium phosphate cement	Calvarial bone defect	[208]
	mRNA	hBMP-2	Cationic liposomes	Collagen	Femoral defect	[209]
	DNA fragment	BMP-7	Adenovirus	Hydroxyapatite, PPF/TCP, PLA Sponge, fibrin gel	Skeletal tissue engineering	[210]
	CRISPR/Cas9 LncRNA H19	YAP	TSPCs-sEVs	Sodium alginate	Tendon tepair	[211]
	CRISPR/Cas9	VEGF	PDA-coated PCL NF	SA-HA	Wound healing	[212]
	siRNA	MMP9	TA	PVA, HLC, TA	Diabetic chronic wounds	[213]
	siRNA	PHD2	2-DMAEMA, 2-PAA, BMA	PTK-UR	Diabetic wound healing	[214]
	siRNA	Ckip-1	Chitosan	Ti	Osseointegration in the osteoporotic condition	[215]
	miR-146a-5p	ERK/Akt	PEI-MSN, YIGSR	Gelatin sponge	Angiogenesis	[40]
3D-printed porous braided scaffolds	miR-23a-3p	PTEN/AKT	hUC-MSCs-sEVs	Gelma/nanoclay	Vascularized bone regeneration	[216]
	miR-210	VEGF	Calcium–silicon nanosphere	β-tricalcium phosphate	Calvarial bone defect	[129]
In vivo 3D print	pDNA	PDGF-B, BMP-2	Chitosan-NPs	Chitosan, collagen	Calvarial bone defect	[83]
Others	miR-29b	Wnt	CMCS	Titanium alloy	Tibial defects	[217]

Annotation: PELCL = poly(ethylene glycol)-b-poly(l-lactide-co-ε-caprolactone), PCL = polycaprolactone, PCLEEP = poly(caprolactone-co-ethyl ethylene phosphate), PEI = polyethyleneimine, HA-GMA = hyaluronic acid-glycidyl methacrylate, Star-PLLs = star-shaped poly(L-lysine) polypeptides, CHA = collagen-hydroxyapatite, PPF/TCP = polypropylene fumarate/tricalcium phosphate, PLA = poly-lactic acid, TSPCs = tendon stem/progenitor cells, PDA = polydopamine, SA = sodium alginate, TA = tannic acid, PVA = poly(vinyl alcohol), HLC = human-like collagen, 2-DMAEMA = 2-(dimethylamino) ethyl methacrylate, 2-PAA = 2-propylacrylic acid, BMA = butyl methacrylate (BMA), PTK-UR = porous poly(thioketal-urethane), and CMCS = carboxymethyl chitosan.

**Table 3:** Designs and applications of sheet-like delivery systems.

Design	DNA/RNA	Target Sites/ Additive Fragments	Vectors	Scaffold Materials	Application	Reference
Bioactive matrix films	miR-126-3p	N/A	SMSCs-exo	Chitosan	Heal full-thickness skin defects	[52]
	siRNA	Smad3	G5-GBA, GelMA	HA	Prevention of peritendinous adhesion	[223]
	pDNA	VEGF165, Ang-1	PEI	Cationized Bombyx mori silk fibroin	Dermal tissue regeneration	[224]
	pDNA	VEGF165, Ang-1	N, N, N-trimethyl chitosan chloride	Collagen, chitosan, silicone membrane	Skin regeneration	[225]
	lncRNA H19	PI3K-Akt	Extracellular vesicle-mimetic nanovesicles	Alginate	Diabetic wounds	[226]
Polymer fiber films	pDNA	ANG	PEI-CMCS	PLGA/CNCs	Skin regeneration	[227]
	pDNA	C-Jun	Chitosan-graft-polyethyleneimine	Polyglutamic acid-coated polylactic acid/silk fibroin parallel fiber film	Nerve regeneration	[228]
	pDNA	BMP-2	Chitosan	PCL	Calvarial bone defect	[86]
	pDNA	VEGF, bFGF	Calcium phosphate nanoparticles	Poly(DL-lactide)-poly(ethylene glycol)	Regeneration of mature blood vessels	[229]
	siRNA	FKBPL	Arginine-rich amphipathic peptide	Alginate/poly-(vinyl alcohol), chitosan/poly-(vinyl alcohol)	Wound repair	[69]
	siRNA	ERK2	PEI-PBA	Polyethylene glycol (PEG)-based polyester	Tendon healing	[100]
Layer-by-layer films	siRNA	CTGF	N/A	Ethicon 4-0 Perma-Hand Silk suture	To reduce cutaneous scar contraction	[230]
	siRNA	TGF-β	N/A	Chitosan, sodium alginate	Excisional wound healing	[231]

Annotation: SMSCs = synovium mesenchymal stem cells, G5-GBA = generation 5 polyamidoamine, GelMA = gelatin-methacryloyl, HA = hyaluronic acid, PEI = polyethyleneimine, CMCS = carboxymethyl chitosan, PLGA = poly(D, L-lactic-co-glycolic acid), CNCs = cellulose nanocrystals, and PCL = polycaprolactone.

**Table 4:** Scaffold Platform Comparative Matrix.

Scaffold Type	Fabrication Method	Nucleic Acid Loaded	Release Mechanism	Advantages	Limitations	Applications
Injectable Hydrogels	Self-assembly, Michael addition, ionic cross-linking	miRNAs, siRNAs, pDNA	Stimuli-responsive (pH, enzyme, temperature, light), swelling, degradation	Minimally invasive, conforms to irregular cavities, localized delivery	Limited mechanical strength, potential premature gelation	Myocardial infarction, diabetic wounds, osteoarthritis
3D-Printed Porous Scaffolds	Direct Ink Writing (DIW), Digital Light Processing (DLP)	miRNAs, pDNA	Porous structure diffusion, scaffold degradation	Tailored pore structure, high mechanical strength, precise geometry	Complex fabrication, limited cell penetration in small pores	Bone regeneration, spinal cord injury
Non-Printed Padding Scaffolds	Lyophilization, chemical cross-linking	pDNA	Scaffold degradation, coating dissolution	Good biocompatibility, fills irregular defects	Insufficient mechanical strength for large/load-bearing bones	Bone tissue formation
Fiber–Hydrogel Composites	Electrospinning, embedding fibers in hydrogel	miRNAs, neurotrophic factors	Sustained release via hydrogel degradation	Mimics native tissue structure, promotes cell alignment and growth	Complex preparation process, higher cost	Spinal cord repair, vascular tissue engineering
Sheet-Like Hydrogels	Casting, cross-linking	miRNAs, siRNAs	Swelling, degradation, stimuli-responsive	Suitable for superficial wounds, provides physical barrier	Limited to shallow tissues, potential scarring issues	Skin wound healing, diabetic ulcers
Polymer Fiber Sheets	Electrospinning	pDNA, siRNAs	Enzyme degradation, fiber breakdown	Large surface area, supports cell growth	May restrict cell penetration, limited to specific cell types	Nerve repair, skin tissue engineering
Layer-by-Layer Thin Sheets	Alternate adsorption of charged materials	siRNAs, growth factors	Layer-by-layer degradation, electrostatic interaction disruption	Precise control of thickness/composition, protects nucleic acids	Complex fabrication, limited to superficial applications	Wound healing, epidermal repair
Host–Guest Self-Assembling Hydrogels	Host–guest molecular recognition (β-CD and AD)	miRNAs	Hydrophobic interaction disruption, shear-thinning	Self-healing, shear-thinning property for injection	Limited by host–guest ratio and affinity, difficult precise regulation	Localized drug delivery in various tissues
Peptide Self-Assembling Hydrogels	Peptide self-organization (e.g., RAD sequence)	Nucleic acids (via electrostatic/hydrogen bonds)	Peptide chain breakdown, electrostatic interaction disruption	Biocompatible, mimics ECM, promotes cell adhesion	Susceptible to enzymatic degradation, limited mechanical strength	Minimally invasive surgery, rapid tissue repair

## Abbreviations

The following abbreviations are used in this manuscript:

PEG	Polyethylene glycol
MSNs	Mesoporous silica nanoparticles
α-CD	Alpha-cyclodextrin
PAMAM	Poly(amidoamine)
HA	Hyaluronic acid
PBA	Phenylboronic acid
OG	Oxidized dextran
ADH-GCA	Adipic acid dihydrazide-grafted catechol-coupled gelatin
ADSCs	Adipose-derived stem cells
hUC-MSCs	Human umbilical cord-mesenchymal stem cells
GelMA	Gelatin methacryloyl
BMSCs	Bone marrow mesenchymal stem cells
CSMA	Chitosan-methyl methacrylate
CNPs	Cerium oxide nanoparticles
SBMA	Chitosan-methyl methacrylate
SBMA	[2-(methacryloloxy) ethyl]dimethyl-(3-sulfopropyl) ammonium hydroxide
HEMA	2-Hydroxyethyl methacrylate
PEGDMA	Polyethylene glycol dimethacrylate
LNPs	Lipid nanoparticles
SA	Stearylamine
Chol	Cholesterol
MeGC	Methacrylated glycol chitosan
DMAEMA	Dimethylaminoethyl methacrylate
PLA	Poly(lactide)
DM	Dimethacrylate
DEGMA	Dimethylaminoethyl methacrylate
PNIPAAm	Poly(N-isopropylacrylamide)
BSA	Cationic bovine serum albumin
PF-127	Pluronic F127
PEG-PLGA-PNIPAM	Poly(ethylene glycol)-b-poly(lactic-co-glycolic acid)-b-poly(N-isopropylacrylamide)
PLGA	Poly(lactic-co-glycolic acid)
PEI	Polyethylenimine
bPEI	Branched polyethylenimine
Dex-PCL-HEMA/PNIPAAm	Dextran-poly(e-caprolactone)-2-hydroxylethylmethacrylate-poly(N-isopropylacrylamide), CBMA=3-[[2-(methacryloyloxy)ethyl] dimethylammonio] propionate
dECM	Decellularized extracellular matrix
EC-Exos	Endothelial cell-derived exosomes
HB-PEGDA	Hyperbranched polyethylene glycol diacrylate
SH-HA	Sulfhydryl-modified hyaluronic acid
SH-HA-PEGDA	Thiolated hyaluronic acid cross-linked with poly(ethylene glycol) diacrylate
DSPE	1,2-distearoyl-sn-glycerol-3-phosphoethanolamine
TAT peptide	Transactivator of transcription peptide
ELP	Elastin-like protein
HYD	Hydrazide
ALD	Aldehyde
SNAs	Spherical nucleic acids
hAD-MSCs	Human adipose-derived mesenchymal stem cells
PEGS-A	PEGylated poly(glycerol sebacate) acrylate (PEGS-A)
PELCL	Poly(ethylene glycol)-b-poly(l-lactide-co-ε-caprolactone)
PCL	Polycaprolactone
PCLEEP	Poly(caprolactone-co-ethyl ethylene phosphate)
PEI	Polyethyleneimine
HA-GMA	Hyaluronic acid-glycidyl methacrylate
Star-PLLs	Star-shaped poly(L-lysine) polypeptides
CHA	Collagen-hydroxyapatite
PPF/TCP	Polypropylene fumarate/tricalcium phosphate
TSPCs	Tendon stem/progenitor cells
PDA	Polydopamine
SA	Sodium alginate
TA	Tannic acid
PVA	Poly(vinyl alcohol)
HLC	Human-like collagen
2-DMAEMA	2-(dimethylamino) ethyl methacrylate
2-PAA	2-propylacrylic acid
BMA	Butyl methacrylate (BMA)
PTK-UR	Porous poly(thioketal-urethane)
CMCS	Carboxymethyl chitosan
SMSCs	Synovium mesenchymal stem cells
G5-GBA	Generation 5 polyamidoamine
CNCs	Cellulose nanocrystals
